# Polymeric Membrane-Based Systems in Transdermal Drug Delivery

**DOI:** 10.3390/polym18030376

**Published:** 2026-01-30

**Authors:** Laura Donato, Paola Bernardo

**Affiliations:** Institute on Membrane Technology “Enrico Drioli” (National Research Council of Italy), 87036 Rende, Italy; p.bernardo@itm.cnr.it

**Keywords:** transdermal drug delivery, polymers, membranes, patches

## Abstract

Controlled drug delivery systems (CDDSs) are increasingly attracting interest from the scientific community in order to achieve highly precise, customized, and efficient therapeutic treatment of various diseases. The challenge is to develop highly innovative devices and appropriate administration methods in order to reduce side effects and further improve patient compliance. In this context, transdermal drug delivery systems (TDDSs) represent smart tools that permit supplying therapeutically effective amounts of drugs at a fixed time using the skin as the administration route. They are non-invasive and allow for avoiding gastric side effects and first-pass metabolism occurring in the liver. TDDSs have been produced using numerous therapeutic agents and, more recently, also biological molecules. However, it must be highlighted that they are complex systems, and their formulation requires a multidisciplinary approach and expertise in polymer chemistry and materials science. A contribution in this direction is given from the integration of membrane technology with biological and pharmaceutical sciences. The present review deals with a general overview of controlled drug delivery systems. Particular attention is devoted to TDDSs and to the materials used for producing polymeric membrane-based TDDSs with a membrane engineering perspective. It also describes the passive and the most advanced active strategies for transdermal delivery. Finally, different transdermal membrane-based release systems, like patches, mixed-matrix membranes, and imprinted membranes are discussed.

## 1. Introduction

The field of drug delivery is attracting a lot interest from academia and industry as can be deduced by the increasing number of studies focusing on such systems and aimed at developing innovative devices capable of localized, smart, as well as on-demand delivery of single or combined drugs. The most common routes for drug delivery are by oral administration, hypodermic injection, and transdermal delivery [1,2,3]. The continuous growth of the pharmaceutical and materials sciences, together with the scientific research on microelectronic engineering and membranes, has allowed for a rapid advancement of controlled delivery technology. The desired aspect is a controlled release profile, keeping the blood concentration of the drug within the therapeutic window, thus enhancing therapeutic effectiveness and reducing systemic toxicity. In this context, the concept of controlled drug delivery (CCD) has given a significant stimulus to the advancement of research in this field. It refers to the possibility of administering an active pharmaceutical ingredient (API) under temporal- and/or spatial-controlled conditions for providing the patients with the efficacy concentration into the body or in a specific area of it within the therapeutic window [1,2,3]. This allows for a reduction in administration frequency and overcomes the problem of rapid or limited release characterizing traditional dosage forms, and therefore avoids/reduces side effects and improves the patients’ compliance and life quality. Resourceful controlled drug delivery systems do not undergo the burst effect, which is characterized by too high release in a short time and can be hazardous for the patients [4,5]. The properties of drugs, like molecular weight, lipophilicity, ionization, and aqueous solubility, are relevant factors influencing their possible use in controlled release formulations. Some typical classes of drugs employed in CDD are antibiotics, anti-inflammatories, antidiabetics, antifibrotics, etc. The development of biocompatible materials made an important contribution in developing these systems, owing to the possibility of producing release matrices based on polymers and membranes [1,6].

The different types of controlled drug delivery devices include oral and injectable systems, implantable systems, transdermal delivery systems, dental and ocular systems, vaginal and uterine systems, and targeted delivery systems [1,2,3,4,5].

**Figure 1:**
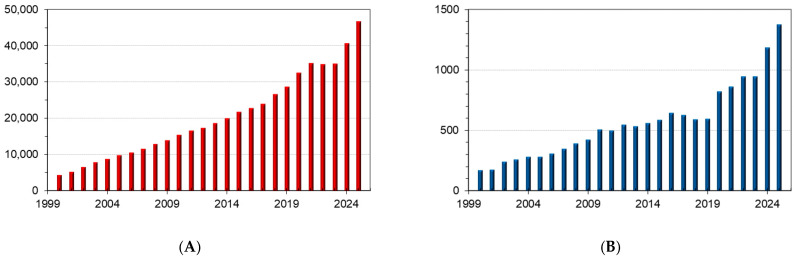
Number of publications by year in the 2000–2025 period. (**A**) “drug delivery”; (**B**) “transdermal drug delivery”.

Over the 2000–2025 period, 530,688 publications have been published according to the search “drug delivery” on the journals indexed in the Scopus database (www.scopus.com accessed on 30 November 2025) and 14,767 studies can be found by performing a search on “transdermal drug delivery” (Figure 1).

Transdermal Drug Delivery Systems (TDDSs), which are formulations or devices that distribute therapeutically effective amounts of drugs using the skin as the administration site, respond to the requirements of possibility of self-administration and easy termination of drug therapy. The drug has to permeate through the skin to be absorbed into the systemic circulation and distributed into the rest of the body [5]. TDDSs are suited for the transport of low-molecular-weight active components which cannot resist the aggressive conditions in the gastrointestinal tract and/or are exposed to substantial first-pass metabolism by the liver. These devices present important benefits that improve patient compliance as a prolonged therapeutic effect, reduce side effects, and improve bioavailability.

The last few years are characterized by a growing geriatric population that suffers from several chronic diseases (e.g., hypertension and orthopedic diseases). In this scenario, transdermal drug delivery patches are particularly suited to responding to the increased demand for devices coupling efficacy with enhanced safety. Indeed, they are non-invasive and offer the possibility to avoid first-pass metabolism. Up to date, different typologies of TDDSs have been developed with the aim to expand this field of research and produce innovative systems capable of releasing both hydrophilic and hydrophobic drugs [1,5].

Industrial research and the scientific community are making considerable efforts devoted to the improvement of the beneficial effects, biocompatibility, biodegradability and cost effectiveness of these devices, as well as to increase the number of drugs to administer through transdermal routes and to develop novel formulations. TDDSs have been widely developed using numerous therapeutic agents and, more recently, also biological molecules. The global market for TDDSs was valued at USD 83.67 billion in 2025. Moreover, a CAGR of 12.12% is estimated, from USD 93.81 billion in 2026 to USD 234.36 billion by 2034 [7].

TDDSs have a complex formulation in which polymers are the core since they provide a matrix to embed bioactive molecules as well as nanocarriers and enhancers [8]. Their design is highly multifaceted and requires interdisciplinarity, particularly expertise in polymer chemistry and materials science.

This review focuses on different membrane types for TDDSs, illustrating the materials used in their fabrication and the importance of various parameters to control for the efficiency of the prepared devices, and the most recent efforts devoted to the improvement of their beneficial effects, biocompatibility, biodegradability, and cost effectiveness are presented. Several produced membrane-based transdermal release systems include matrix and reservoir-based membranes (MMMs) and imprinted membranes (IMs). Finally, future challenges and outlooks are highlighted.

Owing to the typical features of all these systems, we think that the present review will contribute to the scientific community developing more and more innovative drug delivery systems, exploiting advanced membrane technology for enhancing their application at the clinical level and assessing customized single or multi-drug therapies.

## 2. Drug Administration and Controlled Drug Delivery Systems

A drug is any chemical substance (other than food) used for prevention, diagnosis, or therapeutic purposes that produces biological effects into the living organism to which it is administered [9].

**Figure 2:**
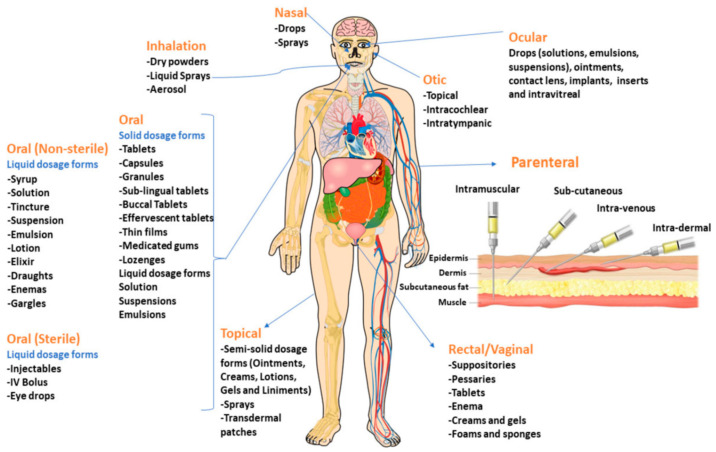
Routes for drug delivery. Reprinted from ref. [1]. Open Access.

The release of a drug into our body, as well as its pharmacokinetics, bioavailability and efficacy, are aspects of increasing interest in the feasibility of therapeutic administration. To date, an enormous number of drugs exists, each one characterized by its own therapeutic properties. Each dosage form is constituted by a combination of a drug/active pharmaceutical ingredient with additives/excipients. The drug possesses therapeutic properties, while the additives/excipients are used for conferring to the formulation of an appropriate shape and stability, as well as for improving bioavailability, safety, taste and patient tolerability [1,10]. Drugs are administered through different ways in view of their solubility and permeability properties, the type of disease, the treatment’s duration and the part/site to be treated. Figure 2 illustrates the most common routes for drug delivery [1]. As is evident, each of them allows for the use of different dosage forms (capsules, drops, gels, granules, ointments, syrups, suspensions, tablets, etc.) depending on the case-by-case necessities.

**Figure 3:**
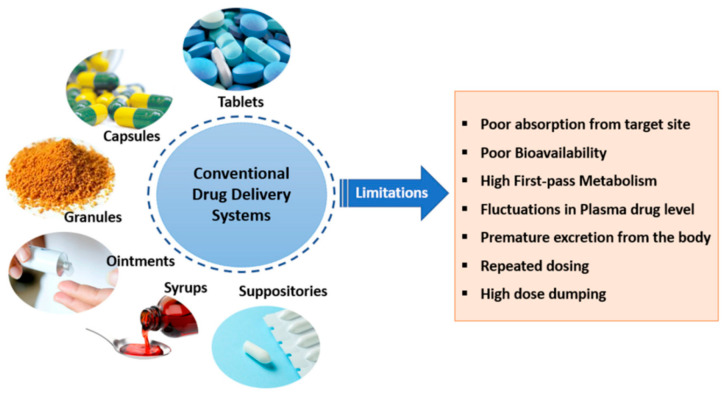
Limitations of conventional drug delivery systems. Reprinted from ref. [1]. Open Access.

Absorption of a drug by our body (and therefore its bioavailability) is influenced by the route of administration, the chemical–physical properties of the drug, any interaction with food and the type of formulation. If the drug concentration in plasma remains below the minimum therapeutic value, its administration will not produce beneficial effects. Conversely, excessive values can be dangerous due to the onset of side effects. So, it is necessary to maintain a balance to achieve the desired therapeutic effect with minimal risk of toxicity. From this viewpoint, conventional administration routes suffer the problem of low drug bioavailability and of the inability to guarantee a sustained release of the administered drug over the desired time with a single dose. Additionally, the administration of multiple doses at regular interval times allows for a fluctuation of plasma concentration that reaches a value outside the therapeutic window. Some other negative aspects are the poor adsorption and bioavailability, rapid excretion, high first-pass metabolism, etc. [1,3,11,12], as shown in Figure 3.

**Figure 4:**
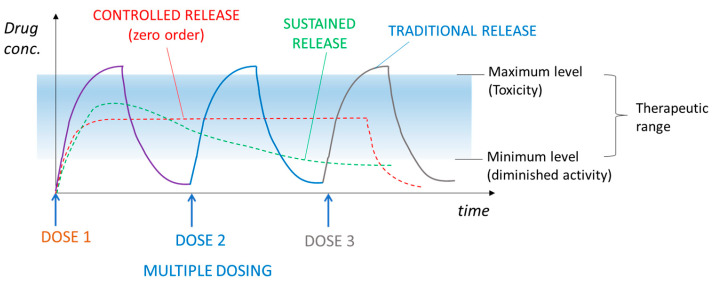
Drug concentration profile during traditional and controlled drug administration.

The advent of the concept of the controlled delivery of a drug provided the possibility of overcoming all these problems. Controlled drug delivery systems (CDDSs) are devices appropriately designed for administering a drug under controlled conditions in order to ensure the maximum therapeutic efficacy and minimal side effects, contrary to what occurs in conventional administration routes. This is because they allow for maintaining a constant drug concentration and remaining within the therapeutic window over the desired time, thus promoting a controlled or sustained release of the drug [3,11,12,13]. Figure 4 shows the profiles of drug concentration in plasma, comparing traditional and controlled drug administration. In the first case, the drug concentration fluctuates at a toxic level and below the minimum effective level. On the other hand, in the case of controlled administration, the drug concentration remains constant in time and within the desired range [13].

Other advantages of CDDSs are a reduction in administration frequency and in the drug amount and accumulation, the achievement of adequate targeting, an enhancement in patient compliance, as well as the potential for tailored medications [1,3,6].

To date, several types of CDDSs have been developed, each one characterized by proper advantages and disadvantages, applications, and release mechanisms. They include oral and ocular systems, implantable and injectable systems, inhalation and rectal systems, transdermal release systems, vaginal and intrauterine systems, and targeted delivery systems [2,3,10,11,14,15]. Drugs used for producing these release devices need to have good stability, solubility, proper molecular weight, a therapeutic window within a specific range useful for avoiding toxicity, biocompatibility with the delivery system, and regulatory requirements [3]. Some typical classes of drugs employed in the formulation of CDDSs are antibiotics, anti-inflammatories, antidiabetics, antifibrotics, etc. [1,6].

The development and improvement of CDDSs faces some obstacles due to the complexity of formulation and production, regulatory aspects, costs and patient variability. Other disadvantages are gastrointestinal resistance in the case of oral dosage forms, interpatient variability, and less flexibility in dosage correction [16].

However, the exploitation of biocompatible materials as well as the combination of pharmaceutical, biological, and materials sciences, as well as polymer and membrane technologies, allowed for obtaining administration of a drug under an expected release kinetic [3,10]. Furthermore, field studies have led to a gradual improvement of these systems. In this context, membrane-based controlled delivery systems proved to be capable of ensuring the desired release profile and the consequent rational therapeutic efficacy of drugs. They are produced with natural and synthetic polymeric materials through their combination or via the incorporation of carriers. According to the drug release mechanism, these systems are classified as follows:

(1) Dissolution controlled;
(2) Diffusion controlled;
(3) Water penetration controlled (osmotic pressure controlled and swelling controlled);
(4) Chemically controlled;
(5) Nanoparticle-based systems [1,17].

In dissolution controlled delivery systems, the drug is coated with or encapsulated in a low soluble polymeric membrane (reservoir systems) as well as dispersed in a polymer matrix (matrix systems). The release is dependent on the dissolution rate of the polymeric material [1,17,18].

In diffusion controlled systems, the drug can be contained in the core of a thin film of a water-resistant (porous or not) and covering polymeric membrane (reservoir system) as well as dissolved or uniformly dispersed through the membrane matrix (matrix system). Drug release is governed by Fick’s diffusion law. The rate limiting step is the diffusion of the drug through the membrane [1,17,19].

In water penetration controlled systems, the drug is released via osmosis or swelling. In the first case, transport occurs thanks to the action of an osmotic pump. In more detail, the drug is located in a nucleus delimited by a semi-permeable membrane provided with an orifice. The exposure of the system to an aqueous liquid leads to water passing through the membrane, dissolving the core and stimulating drug leakage through the orifice at a constant rate [1,17,20,21,22]. In the case of a release occurring via swelling, the drug is dispersed or dissolved in a hydrophilic glassy polymeric matrix. The contact with water molecules/biological fluids determines the solvent penetration in the matrix, the subsequent swelling and the consequent outer slow drug diffusion owing to the change of a polymer from a glassy to a rubbery state [1,17,23].

In chemically controlled delivery systems, the drug is dispersed/dissolved in a biodegradable polymer or conjugated with it. In the first case, the release occurs upon the biodegradation (via erosion) of the polymer under physiological conditions, while in the second case, the release is promoted by the cleavage of polymer–drug bonds (covalent and grafting). Erosion is promoted by chemical or enzymatic reaction and can affect the polymer surface or the bulk. Adequate control of the degradation rate allows for the avoidance of the burst effect typical of systems with quick dissolution [1,17,24,25,26].

Starting from the first generation of CDDSs based on the above-cited release mechanisms, over the last few decades, there has been a prodigious expansion of controlled-release dosage forms and of the number of drugs administered with these systems. The first important goal is a reduction in administration frequency, therefore maintaining a constant concentration throughout the body and finding an equilibrium between the administered and the excreted amount of the drug. Their appropriate formulation permits overcoming some limitations and requires accurate consideration of different aspects, such as the type of disease, a more adequate administration route, a therapeutic dose, the part of the body that needs to be treated, the dosage form, the nature and physiochemical properties of the drug and the polymer-forming membrane, and membrane morphology and thickness [27].

## 3. Release Kinetics and Mathematical Models

Several studies investigated the release rate of drugs in different matrices, evaluating the performance of these systems in vitro and in vivo and contributing to identifying the conditions that generate an initial burst release of the drug or a more controlled release profile [28]. These data are essential to develop mathematical models for characterizing drug release and apply them to the design of real systems [29,30,31]. Several mathematical models are available [32,33] for describing the different mechanisms that can be also present together in a single delivery system (Table 1).

## 4. Transdermal Drug Delivery Systems (TDDSs)—A General Overview

Among the different administration routes, transdermal delivery is a fruitful approach that represents a powerful alternative to traditional oral administration and injections. This section will discuss transdermal drug delivery systems (TDDSs) as well as their properties and applications.

TDDSs are non-invasive tools that permit the distribution of therapeutically effective amounts of drugs using the skin as the administration site at a fixed time [42,43]. Compared to conventional oral dosage or parenteral forms, transdermal drug delivery is a viable administration route with many advantages that improve patient compliance [5,43,44,45]:

- easy, non-invasive, and pain-free administration;
- bypasses the gastrointestinal tract and avoids drug degradation due to the hostile environment of the stomach, as well as a reduction in gastrointestinal side effects. In fact, drugs can be released without interfering action of pH, enzymes and bacterial flora;
- avoids the first-pass hepatic metabolism and reduces the risk of liver dysfunction,
- overcoming the problem of power solubility of drugs in the intestinal fluids;
- promotes a sustained release of the drug of interest into systemic circulation for a long term.

Moreover, drug input can be easily terminated by removing the transdermal patch.

**Figure 5:**
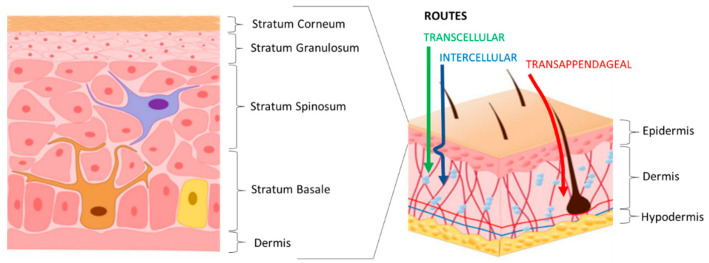
Skin section evidencing the layered skin structure (in epidermis: purple cell = Langerhans cell, brown cell = Melanocyte, yellow cell = Merkel cell) and the skin penetration routes (transepidermal and transappendageal). Adapted from ref. [51], Open Access.

Some important conditions must be evaluated in developing TDDSs: the polymeric material must be suitable for the desired formulation, the drug must be stable and have a short half-life, must be possibly unreactive, and administered for a long time at a constant rate and few mg/day. Furthermore, drug loading should not change the mechanical properties of the polymeric systems. Moreover, the appropriate molecular weight of drugs is in the range of 100–500 Da and, in any case, less than 1000 Da [43,46,47]. Indeed, the therapeutic agents released from these systems have to permeate through the skin to be absorbed into the systemic circulation and distributed to the rest of the body [5,44]. The skin, which covers a surface area of 1.5–2 m^2^, has a layered structure, as evidenced in Figure 5. This feature has to be taken into account in developing TDDSs in order to achieve the desired drug penetration through the skin in adequate amounts to maintain therapeutic levels [48]. In particular, the rigid *stratum corneum* (SC) is the outermost layer that acts as an effective barrier [49]. Its dense structure consists of layers of cornified keratinocytes with lipids contained in the intercellular spaces [50].

The potential pathways of drug diffusion through the skin are depicted in Figure 5. The most prevalent is the transepidermal one that comprises intercellular and transcellular routes. The intercellular path is more tortuous. The transappendageal route involves drug penetration through skin appendages (e.g., hair follicles and sebaceous glands), thus directly bypassing the barrier of the *stratum corneum*. The appendages can be exploited to achieve the passage of larger and more complex molecules, expanding the range of exploited drugs, including polar/ionizable drugs, which are hindered by the lipid-rich skin barrier [48].

Considering the anatomic structure of the skin, the main interest in developing TDDSs is to have appropriate formulations in order to overcome the barrier of the SC and of the vascular system in the dermis for delivering an adequate amount of the drug [43,52]. For most drugs, except those that are highly lipophilic, the rate-determining step for their transport across the skin is located in the SC. However, to reduce inter-individual variability, it would be better to have a rate-controlling step within the delivery device.

Diffusion is the primary mode of transportation in TDSSs [53]. Therefore, most of the models used to reproduce the experimental release data are based on Fick’s diffusion equation [54].

Different strategies are exploited for enhancing drug delivery through physical and chemical methods. These approaches lead to increased drug solubility and its diffusion rate through the skin [43,52,55].

**Figure 6:**
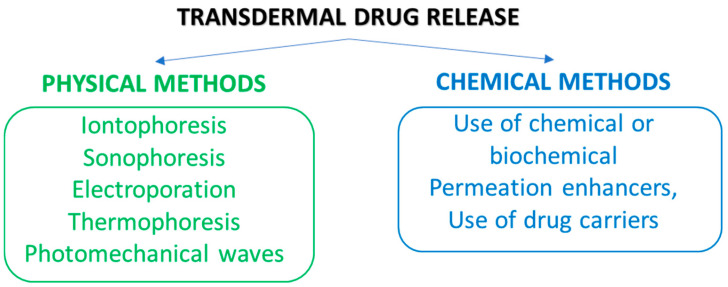
Representative techniques of transdermal drug delivery for enhancing drug transport through the skin.

Physical approaches involve electrical, mechanical and physical stimuli. Some of them are iontophoresis, sonophoresis, electroporation, photomechanical waves, and thermophoresis (Figure 6) [43,52]. Iontophoresis entails the application of a small electrochemical potential gradient for promoting the movement of the drug (in ionic form) across the membrane. Sonophoresis employs ultrasonic waves at low frequency (20–16 MHz) for creating passages (thanks to the phenomenon of cavitation) that facilitate the diffusion of the drug. Also, they determine the increase in local temperature and in the consequent drug penetration [56]. In the electroporation, the application of an electric field determines a pronounced movement of molecules and the drug can trail the hair follicle pathway for passing through the skin, or alternatively, it can cause pore creation. Photomechanical waves can penetrate the skin and create channels available for the drug’s passage. Their application produces only limited ablation with respect to electroporation, which produces a less conservative direct ablation of the skin [43,52,55]. Thermophoresis or thermal ablation consists of a selective disruption of the SC structure by applying a high temperature (above 100 °C). This process results in heating and vaporization of keratin with the creation of microchannels in the skin [43]. Microneedles are minimally invasive novel systems in which the transdermal delivery of drugs occurs through micro-sized needles that punch the skin, allowing for drug release into blood capillaries [44,57,58,59]. Up-to-date microneedles are fabricated by exploiting different strategies (e.g., photolithography, 3D printing, laser-mediated techniques and two-photon polymerization) and exist as solid microneedles, drug-coated microneedles and dissolving needles [43,56]. The ability of microneedles to enhance the permeability of the *SC* of the skin without determining fractures or deformations renders them greatly attractive. Different from hypodermic injections, they create a smaller risk for disease transmission, producing minimal waste [60,61]. Needleless jet injectors are capable of delivering a drug through the skin barrier without a needle by using a high-pressure power source (e.g., spring or compressed gas) [52]. As a result, the skin membrane is disrupted with the formation of micro-holes available for drug transport.

Chemical methods comprise the use of chemical/biochemical permeation-enhancer agents as well as drug carriers. Chemical enhancers are used for inducing structural modifications in the skin by interacting with the lipids and proteins that compose the SC, depressing its barrier role and enhancing drug permeation across the skin [62,63]. These compounds will be discussed in a separate section.

Advanced chemical strategies rely on the use of different typologies of organic or inorganic carriers/modulating agents at the nanoscale level.

## 5. Materials for Producing TDDSs

### 5.1. Polymeric Materials

Polymers are the most important components of TDDSs, contributing to the release and permeation characteristics of drugs, as well as providing the necessary mechanical resistance to formulations [14]. Polymeric films used for producing patches should be non-toxic, stable, non-irritating for skin, easily manufactured, and compatible with the drug and the other components of the device. Their physiochemical properties and structure control the rate of the drug released from the patch. Therefore, transport of drugs through and from the polymeric matrix can be affected by changing the crystallinity and ramification degree of polymeric chains, the swelling degree, and leaching [64]. Other required features of the polymeric material are biocompatibility and good chemical and mechanical stability. The choice of the drug to be administered via the transdermal route depends on its physicochemical and pharmacokinetic features, as well as on the properties of the polymer matrix and on their mutual interaction. Permeation enhancers and plasticizers can affect, in a significant way, the permeability and wearing properties of transdermal patches [65].

**Figure 7:**
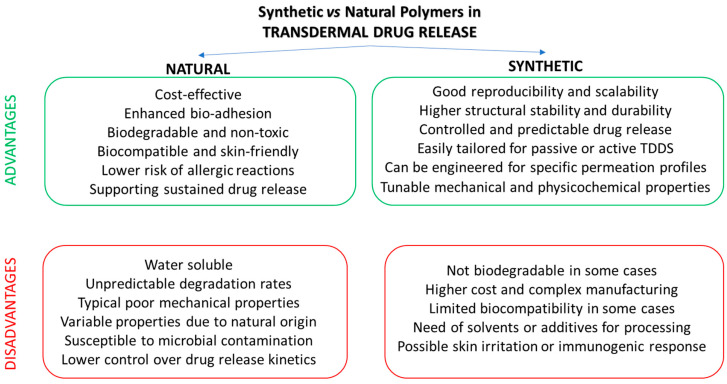
Comparison of natural and synthetic polymers in TDSS applications.

It is important to note that changes in formulation can alter the solubility of the drug in the release medium and its bioavailability. The dispersion of active pharmaceutical ingredients (APIs) within a polymeric matrix when the drug loading is above its crystalline solubility in the polymer produces so-called amorphous solid dispersions (ASDs), thermodynamically metastable binary composites with enhanced bioavailability, and increased dissolution profiles of poorly water-soluble drugs [66]. Upon contact with an aqueous medium, the amorphous drug readily dissolves at a faster rate compared to its crystalline form. In general, TDDS devices require a balance in mechanical strength, drug release and adhesion. Concerning bioadhesion, several polymers can be used in bioadhesive formulations. Representative examples are synthetic materials, such as acrylic derivatives and carbopols; semi-synthetic polymers, like cellulose derivatives and chitosan (CS); or natural polymers, such as alginates and pectin. The most recent trend in materials science is to replace fossil-based polymers with a more sustainable alternative with a reduced impact on the environment and on health. Natural polymers are attractive options for transdermal use due to their excellent biocompatibility and low toxicity that minimize skin irritation [67,68]. Among them, polysaccharides, such as cellulose; chitosan and their semi-synthetic derivatives, alginate, pectin, carrageenan, gum arabic, and guar gum, are natural polymers derived from plants, animals and microbial sources. They are suited for applications in pharmaceutical dosage forms (topical and TDDSs) due to their general recognition as safe (GRAS) materials [69]. Chitosan is the second most abundant natural polysaccharide following cellulose. It is largely applied in the biomedical field in wound dressings and drug delivery, owing to its biodegradability, biocompatibility, and non-toxicity towards human cells. Alginates are anionic polysaccharides that are obtained from the cell walls of brown seaweeds. They are widely used in the pharmaceutical, cosmetic and food industry due to their excellent biodegradability and biocompatibility combined with mucoadhesive properties. However, natural polymers present different limitations, such as microbial contamination, low mechanical strength, poor stability, and reduced viscosity during storage [70]. Moreover, batch-to-batch variations result in dissimilar physicochemical properties and purity, thus causing a difference in drug release profiles [71]. Therefore, to enable pharmaceutical applications of polysaccharides, stringent quality control of natural polysaccharides is required [71]. On the other hand, to improve the performance of natural polymers, crosslinking, grafting, or blending with other polymers are successful strategies [72,73]. A critical comparison of the properties of synthetic or natural polymers in TDSS applications is provided in Figure 7. Furthermore, Table 2 reports the most common polymeric materials adopted for producing TDDSs according to their origin (synthetic, semi-synthetic and natural materials), highlighting their most distinctive features.

### 5.2. Chemical Enhancers

Different types of chemical enhancers are exploited in TDDS formulations in order to regulate drug permeation rates through the skin. Table 3 summarizes the most investigated chemicals.

In general, hydrophobic molecules tend to penetrate more easily into the lipid bilayer. However, the shape and molecular weight of a chemical enhancer influence its activity in reducing the barrier function of the skin’s outer layer. Smaller terpenes are more active sorption enhancers than larger terpenes [62]. The longer the alkylic chain on alkyl-substituted pyrrolidone derivatives, the better the enhancement [137]. Cyclic compounds typically display a better enhancement ability [138]. Moreover, to understand the mechanism of action of chemical enhancers, chemical modeling can be useful (e.g., evidencing their interaction with the bilayer interface of the skin [139]).

However, the use of chemical enhancers can pose some issues. The chemical enhancement process is concentration-dependent, requiring, in some cases, high concentrations. Some chemicals can display toxicity or promote skin irritation and allergy when used at high concentrations [62]. A representative example is the organic solvent dimethyl sulfoxide (DMSO) that requires high concentrations to displace water surrounding the polar head groups of intercellular lipids of the SC, leading to reduced packing of the lipid hydrocarbon tails [140]. Moreover, the efficient concentration for each chemical enhancer changes with the type of drug used [141]. Therefore, many studies are focusing on the use of bioenhancers/natural herb ingredients [132,133]. They are used as is or combined with drugs for providing synergistic therapeutic effects owing to their own therapeutic properties [42]. However, their appropriate choice is not immediate and one needs to be cautious, owing to their biological features and the possible interaction with the formulation components. They include complex polysaccharides, herbal extracts, essential oils, fatty acid esters, terpenes and terpenoids. Essential oils are volatile compounds usually used for stimulating the absorption of pills applied on the skin from topical formulations and stimulating wound healing. Owing to their numerous properties, like antioxidant, anti-inflammatory and anticancer activities, as well as antibacterial, antifungal and antiviral activity, they are used in cosmetic, food, medicinal and pharmaceutical fields. Terpenes are a great class of hydrocarbon compounds and their derivatives are responsible for the scents and aromas of plants and flowers. They are regarded as safe (GRAS) compounds and are superior to other traditional enhancers. They exhibit low toxicity, reduced irritancy, and acceptable penetration enhancement. Other examples of natural permeation enhancers are fatty acids and herbal extracts. The addition of fatty acids to a TD formulation allows for the achievement of high flux through the skin and the absence of irritation. A recent example of the use of oleic acid as an enhancer is the development of transdermal gel formulations for the sustained release of raloxifene, a selective estrogen receptor modulator that is approved for the prevention of invasive breast cancer [142]. In vitro results show the potential of reservoir-based TDDSs for the weekly administration of this drug.

In the framework of herbal ingredients, it has been proven that some flavonoids used as enhancers penetrated deeply into the skin layer, resulting in their being also useful for topical delivery [42,132,133].

Biochemical enhancers are represented by macromolecules that are able to be broken down or to pass through the skin barrier in the stratum corneum. Two examples are polyarginine, which is capable of reaching the dermis layer, and the pore-former magainin. Biochemical enhancers are not used very much, but they could prove very useful in new strategies for developing advanced formulations.

The use of Ionic Liquids (ILs) represents an innovation in the TDDS field [134,135,136]. ILs are salts typically constituted by large organic cations and inorganic anions with a low vapor pressure [143]. By playing with the chemical structure of the ionic constituents, ILs display enhanced tunability and versatility for various applications, including separation processes as extraction media, in catalysis, and in materials science as interfacial agents for nanoparticles in nanocomposites [144]. ILs are considered for pharmaceutical applications [136,145].

Deep Eutectic Solvents (DESs) are emerging as new eco-friendly permeation enhancers. They consist of a mixture of two or more substances in an appropriate ratio in order to achieve a great reduction in the melting point near room temperature. This is due to the occurrence of strong intermolecular hydrogen bonds [146]. Some of their properties are biocompatibility, low toxicity, biodegradability, low costs, easy preparation, high thermal stability, reusability and low volatility. DESs are recognized as greener substituents of traditional organic solvents and ILs in pharmaceutical and chemical processes [146]. In particular, DESs are excellent vehicles able to dissolve a large spectrum of APIs. Among them, combinations of fatty acids/terpenes are cheap, sustainable and tuneable hydrophobic solvents that present moderate viscosity, comparatively lower toxicity, and chemical stability. A DES composed of menthol (MN) and capric acid (CA) was used as a green solvent to enhance solubility and penetration of the antipsychotic risperidone [147]. Eutectic mixtures of MN were also reported as vehicles for transdermal drug delivery as in the case of ibuprofen [148].

## 6. Parameters That Influence Drug Release

A good design of TDDS devices has to consider different parameters that affect the release profile. For example, it is crucial to control the drug distribution and the morphology of the polymeric matrix. A uniform drug distribution in the membrane matrix will be released at a more gradual rate.

### 6.1. Interaction of the Polymer Matrix with the Drug

The successful development of a TDDS is influenced by the appropriate selection of the components of the release systems. In this context, important aspects to consider are the size, molecular weight, and surface chemistry, as well as intermolecular interactions, such as polymer–drug, drug–drug, drug–skin, polymer–skin, etc. [149]. The choice of the polymer for delivering a specific therapeutic agent depends on the physicochemical properties of the drug, its pharmacokinetic properties and its interactions with the membrane.

The interaction of the polymer matrix with the drug molecules during membrane formation has to be considered as well. A study on CA films prepared by nonsolvent-induced phase separation and loaded with naproxen demonstrated that the drug release rate can be controlled by membrane morphology [150]. The CA membranes developed by phase separation displayed consistent and controllable physical properties and more efficient drug release compared to samples prepared using traditional casting techniques. In particular, the presence of drug molecules, even though they are not nonsolvent, can impede the locking-in of the membrane structure. This result is a combination of the drug crystallization rate, and mass transfer path of the casting solution. Interestingly, the burst effect, typically found in film prepared by solvent casting at high drug loading (ca. 40% in this study), can be suppressed in the films produced by phase inversion. In particular, the authors discussed the interplay between phase inversion, drug crystallization and membrane formation [150]. This study showed that the drug naproxen reduced glass transition and led to honeycomb structures with a low resistance to drug transport, evidencing a relationship between the membrane structure with the release profile.

Computational modeling can be relevant for designing and engineering efficient and harmless drug delivery formulations. These approaches can support polymer screening by implementing methods for the assessment of the polymer/drug compatibility and interactions [151]. The prediction of these interactions can help to understand their effect on the drug release rate and on the response of delivery devices. Also, information difficult to obtain experimentally and the reduction in labor time for choosing reagent types and their amount is available. This permits us to identify a good starting point for obtaining the desired release profile of the administered drug along the requested time. So, with the aid of molecular simulation, it is possible to avoid a low or excessive drug concentration in the area of interest, which would be inadequate or harmful, respectively. Additionally, risk for environmental health can be also reduced during the production process. The simulation models (quantum mechanics (QM), molecular dynamics simulation, quantum computing, computer-aided drug designs, machine algorithms, machine learning, etc.) allow for obtaining different levels of information, identifying those best suited to the case of interest [150,152,153,154]. Interestingly, the combination of skin-permeation modeling with computational studies to predict polymer–drug compatibility, miscibility and interaction strength could improve the control of the release profile. This is thanks to the information about the freedom of movement of the drug within the polymer matrix and the possibility to understand the mechanisms involved in skin permeation enhancement. Indeed, the stronger the interactions, the more the drug’s diffusion capacity is reduced [152,155].

AI techniques, such as Machine Learning (ML) models, can accelerate pharmaceutical research [156]. In more detail, Artificial Neural Networks (ANNs) were used as predictive tools for the release of voclosporin, an effective drug for treating ocular diseases, from a polycarbonate matrix to determine the key elements that affect the drug release kinetics [157]. Deep Neural Network (DNN) models were used for enhancing the accuracy in predicting the release of piroxicam from a film formulation based on chitosan, xanthan gum and carboxymethyl derivatives [157]. A Generative Adversarial Network (GAN) was implemented to expand the limited experimental data and to generate reasonable formulations [157].

Apart from chemical interactions, surface morphology, wettability, and crystallinity of the polymer matrix can also affect the performance of a delivery system.

### 6.2. Degree of Swelling

The degree of swelling of the matrix material in water is an important parameter to control the drug release rate. The absorption of water opens up the pores, thus enabling the drug diffusion in the swollen matrix. Blending represents a useful approach to achieve the desired combination of properties for the polymeric matrix. Although PEBAX shows excellent mechanical and physicochemical properties, to improve its poor water absorption capacity, other hydrophilic polymers, like PVA or CS, can be considered [89]. The hydrophilic CA was blended with the more stable PU and loaded with paclitaxel, producing electrospun fibers [158]. An increasing CA amount in the composites results in a higher swelling degree and a larger increase in drug release. The change in the blend composition did not affect the initial release that could be related to drugs that are located at the nanofiber surface. Once these external drug molecules were released, the subsequent release was different depending on the diffusion of the drugs across the matrix.

### 6.3. Degradation/Dissolution Rate

The degradation/dissolution rate of degradable polymer matrices strongly affects the drug release process. Water-soluble polymers such as chitosan, HPMC, PVA, and PVP are key materials for TDDSs, enabling the encapsulation of hydrophilic drugs. Moreover, the use of these polymers to form matrices or hydrogels that swell and dissolve when in contact with skin moisture results in a quick and complete release of the loaded drugs.

Polysaccharides have been investigated by many authors for drug delivery, owing to their hydrogel-forming ability. Gels are semisolid formulations consisting of a liquid phase inside a 3D crosslinked polymeric matrix. The nature of the liquid phase permits us to distinguish different type of gels, such as hydrogels that contain water and organogels that contain an organic solvent. Examples of more recent types are emulgels, bigels, aerogels and so on [44,159,160,161]. Often, a water-soluble component such as PVP is blended with other polymeric materials. PVP was combined with cellulose acetate to fabricate nanofibers able to control the release rate of ferulic acid (FA), an active component with low water solubility [102]. An increasing amount of PVP improved the release of FA. CA fibers take more than 70 hrs to release the last 10% of the drug. On the other hand, the fibers containing 8% of PVP required less than 35 hrs. Indeed, being water-soluble, PVP creates pores in the fiber; thus, the loaded drug is more exposed to the surrounding media, enabling its sustained release. The use of PVP was also considered in the development of PVB-based membranes loaded with quercetin (QCT), which were produced in order to exploit the antioxidant and antibiofilm properties of flavonoids [162]. The presence of a water-soluble polymer (PVP) in the membrane increases the release efficacy in an aqueous medium. PVP was also beneficial to enhance the QCT delivery in an aqueous medium from electrospun membranes based on polycaprolactone [163]. The hydrophilic PVP acts as a diffusion enabler for QCT within polymeric formulations [163].

### 6.4. Cristallinity

A higher crystallinity of the polymer matrix can reduce its degradation rate and solubility. The annealing process can be exploited to this aim. A study on curcumin loaded into electrospun mats based on regenerated silk fibroin (RSF) [164] showed reduced solubility of electrospun RSF in a release medium (phosphate buffer saline (PBS) and ethanol) by water annealing. The increased annealing temperature results in a larger content of crystals that act as impermeable regions, reducing the water penetration in the membrane. Chemical crosslinking is another method to reduce the degradation/dissolution rate of the matrix material since it reduces the number of sites available for interaction with the environment. The release of a topical drug (nicrotinamide) was modulated by crosslinking a blend of water-soluble HEC and PVA [165]. Films based on pristine materials released 80% of the drug in 2 h, while scaffolds that were crosslinked using citric acid reached the same level of released drug in 24 h.

### 6.5. Hydrophobicity

An ideal drug candidate would have sufficient lipophilicity to be transported through the SC. However, a successive partitioning involving the aqueous-viable epidermis and the systemic circulation also require sufficient hydrophilicity.

The wettability of a membrane affects the rate of water penetration in its structure and thus the swelling and the related rate of drug release. The great hydrophobicity of some drugs (e.g., curcumin) strongly limited their topical permeability. A study on PLGA-PU fibers showed that the in vitro release of the more hydrophilic drug (tenofovir) was completed within 30 min, while the hydrophobic drug (levonorgestrel) required 4 h [166]. A sustained release for a highly hydrophobic drug such as curcumin was obtained using hydrophobic Zein-based films, while films based on the hydrophilic PVP showed a sustained release of a less hydrophobic drug (i.e., terbinafine hydrochloride) [167]. In this respect, the morphology and thus the porosity and roughness of a membrane can increase hydrophobicity [168]. According to the Cassie–Baxter model, hydrophobicity of a porous material is due to air pockets within the pores [169]. Porous structures formed by the interconnecting fibers, such as those obtained by electrospinning starting from a material with a poor tendency to adsorb water, are more hydrophobic.

Transdermal patches were formulated by employing a solvent-casting technique with different ratios of hydrophilic polymers (sodium carboxymethylcellulose (CMC-Na) and hydroxypropyl methylcellulose (HPMC)) for the local delivery of methotrexate [104]. A patch formulated with a 1:1 polymer concentration served as the control formulation. An optimized formulation combines CMC-Na and HPMC (5:1).

A blend of hydrophilic and hydrophobic materials can be used to tailor the release properties. Transdermal patches of glibenclamide were developed by mixing HPMC E50 as a hydrophilic polymer and Eudragit RS 100 as a hydrophobic polymer [103]. The solvent-casting technique was employed using chloroform and methanol as the casting solvent. FTIR analysis evidenced the absence of drug–polymer interactions. An increased concentration of Eudragit in the formulation decreased the amount of drug penetration through the membrane.

In some cases, crosslinking treatments improve hydrophobicity as observed for PVA/SA nanofibers lutein-loaded and crosslinked using a mixture of glutaraldehyde and a saturated boric acid solution [101]. The PVA/SA nanofibers keep a hydrophilic character when the duration of the crosslinking reaction was one hour, and drug release is determined by diffusion and dissolution of the polymer in water. Instead, a prolonged crosslinking of up to 5 h rendered the nanofibrous membrane hydrophobic, resulting in diffusion as the main release mechanism.

## 7. Transdermal Patches

Patches are the most representative TDDSs. They are flexible and patient-friendly medicated adhesives that can be directly applied on the skin and easily removed at any time. The role of each component is relevant for ensuring the good performance of the delivery systems and limiting the possibility of incurring in a burst or extremely slow-release kinetics [170]. The heart of their components is represented by polymers, both in the form of porous or dense membrane films loaded with drugs, excipients, nanocarriers, etc. [58,171,172]. All typologies include a protective liner that is removed before using the patch and a backing laminate layer, a drug-impermeable membrane made of elastomers that protects the other layer from the external environment, prevents the drug from leaking out of the system and provides the required flexibility. A pressure-sensitive adhesive enables the adhesion of the patch to the skin and holds the patches’ layers together. In addition, the adhesive layer may include the drug and/or a permeation enhancer. The drug can be supplied from a reservoir or from another part of the patch. Other components of patches are the excipients (enhancers, plasticizers, solvents, and surfactants).

**Figure 8:**
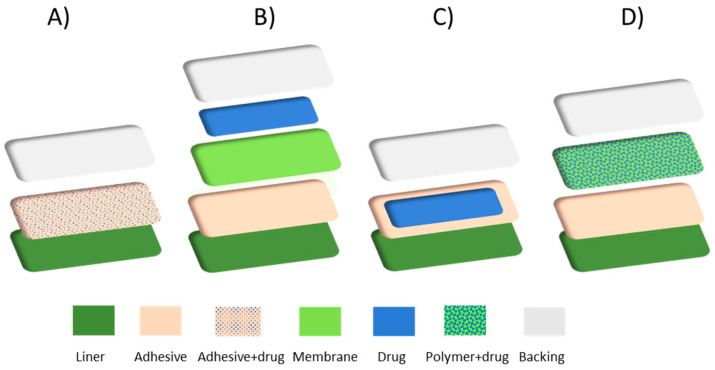
Types of adhesive transdermal patches. (**A**) Single-layer drug-in system; (**B**) reservoir system; (**C**) drug matrix-in system; (**D**) micro-reservoir system.

Today, four different types of transdermal patches are available: (1) drug-in-adhesive systems, (2) reservoir systems, (3) matrix systems, and (4) micro-reservoir systems (see Figure 8). The first one is the simplest. Its core consists of an adhesive membrane film containing the drug and controlling the release rate. In reservoir systems, the drug is contained in a core interposed between the backing layer and a porous membrane; it can be present in dissolved, dispersed, or gel form. Matrix systems are characterized by a uniform distribution of the drug into the polymeric membrane matrix, while micro-reservoir systems represent a combination of reservoir and matrix systems [58,171,172]. Drug-in-adhesive and matrix-type patches are preferentially used rather than reservoir systems. This is due to their simple design and consistent adhesion to the skin [58,170]. Reservoir and micro-reservoir patches, characterized by a more complex formulation, are used in the presence of specific therapeutic objectives.

Physicochemical characterizations, kinetic modeling, in vitro drug release, ex vivo drug permeation, skin drug retention, and in vivo studies are the common methods to evaluate the performance of formulated patches. In 1985, Gale and Berggren developed one of the first patches using an ethylene vinyl acetate membrane loaded with nitroglycerin. The advancement of knowledge in the controlled drug delivery and permeation field allowed for the improvement of the performance of TDDs and the expansion of their application areas [58,170,172]. Table 4 lists some examples of commercially available patches for the treatment of specific conditions or pathologies, such as chronic suffering, stopping smoking, hormone spare therapy, dyslipidemia, etc. [58,173,174]. Representative drugs delivered from commercial patches are atenolol, clonidine, estradiol, fentanyl, nicotine and nitroglycerine [58,170]. Moreover, natural components with bioactive properties (e.g., capsaicin, menthol, caffein, etc.) were recently exploited in several studies [175] as well as in marketed devices [176].

The following types of transdemal patches will be discussed in the next sections:

(1) Matrix type (drug-in polymer);
(2) Nanocomposite membranes;
(3) Molecularly imprinted membranes.

### 7.1. Matrix Type (Drug-In Polymer)

A drug-releasing membrane/matrix presents the drug dispersed into a matrix made of natural/synthetic polymers/elastomers.

Several conventional fabrication methods, such as solvent casting, phase inversion, coating, interfacial polymerization, electrospinning, etc., have been implemented for preparing polymer and drug TDDSs.

Frequently, chemical enhancers are introduced in the polymeric matrix during the fabrication process. For example, a transdermal patch loaded with olanzapine for treating schizophrenia and bipolar disorder was successfully fabricated by formulating a suspension-based TDS in silicone using oleic acid as a chemical enhancer [209]. The patch was non-irritant for the skin and, as desired, released the drug in 3 days.

Matrix-type HPMC-based polymeric films loaded with the antidepressant sertraline hydrochloride were prepared, evaluating the influence of different permeation enhancers (limonene, oleic acid, and Span 80) on the release rate of the drug [210]. A diffusion-mediated release mechanism and zero-order release kinetics were observed for all the produced patches. Permeation tests through the biological barrier of albino mice evidenced, with formulations containing oleic acid as the permeation enhancer, the highest flux, diffusion and permeability coefficients. This was due to the action of oleic acid on the lipids of the *stratum corneum* resulting in an effortless partition in it. Additionally, the drug permeation was enhanced, owing to the formation of polar channels by appropriate solvents, like ethanol [210]. Moreover, other substances, such as polymer plasticizers, can be considered in TDDS formulation to modify the API release rate. Hardainiyan et al. [211] developed transdermal matrix-type patches containing the tricyclic antidepressant imipramine hydrochloride, the plasticizer polyethylene glycol (PEG-400) and different combinations of the hydrophilic polymers HPMC K100M and PVP K-30. The patches were prepared according to the solvent evaporation method. A chloroform/methanol (3:2) mixture was used as the solvent and DMSO as the permeation enhancer. Results of in vitro skin permeation through rat skin show that the release rate of the drug increased by increasing the content of the more hydrophilic polymer. After 24 h, the best formulation (HPMC-PVP 8:2) released 84.71 ± 3.07% of the drug [211]. Different combination ratios of hydrophilic and hydrophobic polymers, including the plasticizer propylene glycol (PG), were used for preparing a transdermal patch by the solvent evaporation technique, investigating their effect on the release features of the β1-receptor selective antagonist atenolol [212]. The used polymeric materials were HPMC, PVP and ethylcellulose (EC). Span 80 was the permeation enhancer and PG was used as the plasticizer. In vitro release studies were carried out using a Franz diffusion cell equipped with an egg semi-permeable membrane as the diffusion barrier (in direct contact with the patch). Results evidence that the formulations containing only the two hydrophilic polymers, HPMC and PVP, exhibited the highest percentage of atenolol release [212].

Transdermal patches loaded with the anti-inflammatory tramadol hydrochloride were prepared using Eudragit RL-100, Eudragit RS-100 and HPMC as the polymer matrix; triethyl citrate or PEG 400 as the plasticizer and DMSO as the penetration enhancer [213]. Eudragit produced crystallization-free patches that facilitate drug transport. Furthermore, triethyl citrate resulted in a better plasticizer than PEG-400 [213]. Transdermal patches for the sustained release of the Class III antiarrhythmic amiodarone were also prepared using HPMC, EC and Eudragit RS 100 as the polymeric matrix; glycerol as the plasticizer and DMSO as the permeation enhancer [214]. Recently, Eudragit^®^ E100 and a copolymer (Kollidon^®^ VA64) were successfully used for fabricating adhesive patches loaded with cannabidiolic acid and tetrahydrocannabinolic acid [215]. These drugs display analgesic, anti-inflammatory, anticancer, anti-spasmodic and psychedelic activities. Succinic acid, dibutyl phthalate, and permeation enhancers such as oleic acid, isopropyl myristate, mixtures of oleic acid and isopropyl myristate, ethoxydiglycol, and sesame oil were added in the proposed formulations. For both drugs, the highest release profile was achieved in the presence of ethoxydiglycol as the permeation enhancer [215].

A novel temperature-sensitive polymer, poly(N-vinyl caprolactam) [P(NVCL)], was used as the polymeric matrix for fabricating patches for the transdermal release of sinomenine hydrochloride, a drug used in the treatment of gouty arthritis [216]. The optimized formulation consisted of 5% P(NVCL), 1.0% glycerol, 1.5% sodium polyacrylate, 4.0% of the drug and three permeation enhancers (3% Azone, 6% borneol and 3% menthol) that acted synergistically, reaching a cumulative rat skin penetration of 248.6 ± 15.7 μg/cm^2^ over 24 h [216]. A reservoir-type patch for the transdermal release of simvastatin was produced using the block copolymer poloxamer 407 and the permeation enhancer D-limonene [217]. In vivo tests carried out for 14 days in hyperlipidemia-induced Sprague–Dawley rats showed an important reduction in the blood lipid profile when a formulation containing 1.5% (*w*/*w*) of simvastatin, 25% (*w*/*w*) of poloxamer 407 and 10% (*w*/*w*) of D-limonene was used [217].

Recently, matrix patches loaded with atenolol were produced combining low- and high-molecular-weight polyisobutene [218]. Ex vivo permeation experiments performed on rat skin showed that formulations with different proportions of polyisobutene affected the release of the drug in a different entity from the adhesive patches. The optimum combination for achieving a sustained release over time was the addition of 200 mg of EC as the thickening agent and a high—molecular-weight/low-molecular-weight ratio of polyisobutenes equal to 3:1. After 30 h, this system allowed for a cumulative atenolol permeation rate of 67.09% relative to the initially loaded drug amount [218].

Dissolving transdermal delivery systems, which are based on biodegradable polymeric materials, facilitate the complete release of the loaded active molecules, owing to their rapid disintegration. Different biodegradable polymers are investigated to develop dissolving devices. Representative examples are PGA, which degrades completely in a few days; PLA, which requires a few months; and PCL, which has a slower degradation rate (a few years). They can be also used in combination. A blend of poly(lactic-glycolic acid) (PLGA) with different lactic-to-glycolic ratios, PLA and PCL was adopted to prepare electrospun membranes with a tailored release rate of the anti-glioma drug temozolomide [219]. A higher glycolic content in the PLGA copolymer produced a burst release of the drugs, probably due to the faster degradation of PGA. Conversely, the presence of the slower-degrading lactic acid diminishes the burst release. Small amounts of PCL in the blend also allowed for control of the burst release. Biodegradable sodium hyaluronate-based microneedle patches were produced for delivering *Dermatophagoides farinae* for inducing an immune response against this allergen in the treatment of atopic dermatitis in mice [220].

**Figure 9:**
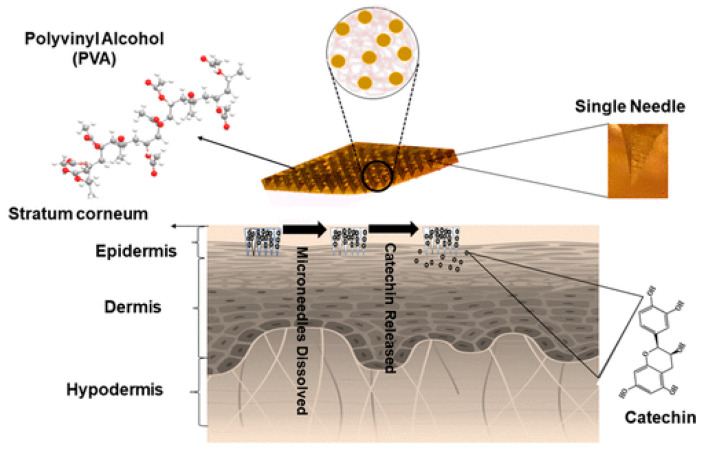
Transdermal delivery of catechin using dissolving poly(vinylalcohol)-based microneedles. Reprinted from ref. [98] with the permission of American Chemical Society, copyright 2023.

The use of dissolving microneedles represents a highly attractive approach for transdermal drug delivery. PVA is an interesting water-soluble polymer and it is used to prepare dissolving microneedle patches. An example is related to the release of catechin (Figure 9) [98]. Other components in the PVA patch were 2% DMSO, 5% carboxymethyl cellulose (CMC), 5% fructose and 5% sucrose. The presence of a sugar made the structure brittle, resulting in reduced drug release compared with sugar-free microneedles. The optimal formulation for the catechin release (86.6%) and swelling ratio (283.8%) was that containing CMC [97].

The above-discussed examples are only a small number of the numerous patches based on the membrane matrix and readers can find more information in the literature [221,222].

### 7.2. Nanocomposite Membranes for TD Drug Delivery

**Figure 10:**
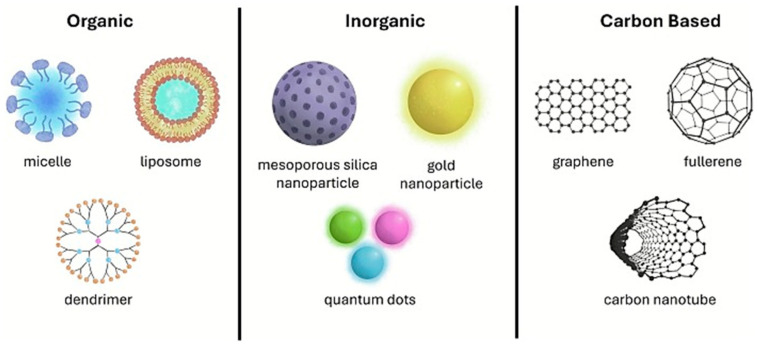
Some nanostructured materials used in TDDS formulations (organic, inorganic and carbon-based nanoparticles). Reprinted from ref. [223]. Open Access.

Nanocomposite membranes are a type of advanced membranes enclosing a discrete phase within the continuous polymer matrix in order to improve membrane performance. The dispersed phase can be represented by organic nanoparticles, inorganic nanoparticles and carbon-based materials (see Figure 10) [223,224].

Organic fillers are non-toxic, biocompatible and biodegradable. However, they exhibit low drug-loading capacity, poor stability, a low reproducibility rate and a short shelf life, which are factors limiting their wide employment. They include microemulsions, nanostructured lipid carriers (NLCs), ethosomes, dendrimers, invasomes, transferosomes, liposomes, solid lipid nanoparticles (SLNs), etc. [52]. Among them, liposomes are one consolidated example capable of encapsulating both lipophilic and hydrophilic drug molecules [44,223,225,226]. They are spherical-shaped vesicles consisting of a hydrous compartment encircled by a phospholipidic bilayer membrane. In more detail, the central aqueous core can encapsulate hydrophilic drugs, while the external lipidic bilayer encapsulates lipophilic drug molecules. In addition, amphiphilic drugs can be positioned at the water/lipid bilayer interface. The ability of liposomes to penetrate the *stratum corneum* and the ultra-small particle size as well as the aptitude to improve the solubility and the bioavailability of drugs render them hopeful TDDSs for treating various diseases, like alopecia, cancer, infection, psoriasis, etc. [227,228,229]. More recent carriers (e.g., polymeric micelles (PMs), solid lipid nanoparticles (SLNs), and nanostructured lipid carriers (NLCs)) are under investigation, and readers can find more detailed information in the open literature [47,52,230].

Inorganic fillers display high stability and loading capacity than organic fillers. Furthermore, they exhibit hydrophilicity and a widespread range of physicochemical, mechanical, magnetic, and optical characteristics as well as the capacity to be modified with ligands [223,231,232]. These features have made them interesting in controlled drug delivery applications. In this context, they have attracted attention as carriage systems for delivering drugs at specific areas of the body, allowing for the improvement of drug bioavailability, solubility into the blood and drug absorption time and efficacy, reduced release times and side effects, and the removal of drug aggregation. Some disadvantages with respect to organic fillers are cellular toxicity, less biocompatibility and low biodegradability [231,232]. Inorganic fillers include different materials, such as aluminum, gold and silver nanoparticles; cesium oxide iron oxide titanium oxide zinc oxide; zeolites; graphene-based fillers and hydroxyapatite [223,231,232].

Even if there are some problems to overcome for their large application, the suitability of nanofillers in exerting different roles, acting as drug carriers, and acting as release-modulating agents for enhancing drug delivery efficacy of release systems has been proven. Additionally, the large pore size of some of them has been exploited for promoting the free release of large-sized drug molecules [233]. Moreover, nanoparticles can add stimuli-responsive properties to patches, as in the case of pH-responsive liposomes and transethosomes prepared using a PVA coating to delay the drug delivery for the transdermal release of nicotinamide mononucleotide that is involved in the production of the coenzyme NAD^+^ [234]. It is important to highlight the possibility of enhancing the performance of natural polymers by introducing selected nanofillers in these matrices. In particular, thermal stability as well as mechanical and barrier properties can be improved in nanocomposites [235]. For example, high-performance PLA bionanocomposites can be prepared by combining PLA with nanoparticles like bio-based reinforcements such as nanocellulose, carbon-based materials and metallic nanofillers [236,237]. The next section will discuss their incorporation into polymeric materials in the production of smart transdermal drug delivery membrane-based systems.

#### 7.2.1. Mixed-Matrix Membranes

The so-called mixed-matrix membranes (MMMs) are nanostructured membranes combining properties of a polymeric matrix with those of inorganic materials (fillers) dispersed into it. In more detail, MMMs consist of an organic polymeric matrix containing inorganic fillers like carbon molecular sieves, silica and carbon nanotubes, and zeolites [238]. They have received great attention, owing to their numerous properties that include antifouling behavior, good permeability and mechanical strength, and selective properties. These features originate from tailor-made and designed structures, which can be controlled during their production by appropriately choosing operating parameters, polymers and fillers dispersed into the polymeric matrix on the basis of the specific application. Thanks to these properties, MMMs are widely investigated in the field of gas and liquid separation [86,238]. However, some studies have evidenced their great potential in biomedical and pharmaceutical applications, including controlled drug delivery [86]. Regarding the different fillers used for their production, zeolites were capable of meeting specific requirements. They are aluminosilicate inorganic materials that have a crystalline structure. Zeolites can have a microporous, mesoporous, or microporous structure, combining different pore sizes in the same structure. Owing to the regular and uniform shape of their pores and their ion-exchange capacities, biocompatibility, large surface areas and manageable physicochemical properties, zeolites are used in the pharmacological area and have attracted attention as potential fillers in developing TDDSs [239,240,241]. 

In this context, Donato et al. applied the phase inversion technique to prepare for the first time polydimethylsiloxane (PDMS)-based MMMs with different amounts of hydrophilic NaX zeolites and drug loading to modulate the release of the opioid tramadol hydrochloride, a drug used for the treatment of pain due to surgery or chronic diseases [86,242]. Data of in vitro release tests show that at 0.2 wt% of drug loading, an increase in the zeolite content from 8 wt% to 17 wt% resulted in a lower and linear release profile than the simple PDMS-based membrane. These results were the consequence of interactions between the hydrophilic zeolite and the polar molecules of tramadol. However, the formation of zeolite clusters, obtained at high zeolite concentration, determined an excessive reduction in the release rate due to their hindering action and to the presence of a more tortuous pathway for the diffusion of tramadol. The released data fitted well with the Higuchi, Bhaskar and Korsemeyer-Peppas models [86]. PDMS-based MMMs containing the NaX zeolite as the filler were also fabricated, aiming at the transdermal delivery of the lipid-lowering drug gemfibrozil [243]. In another study [85], PDMS was used for preparing ibuprofen-loaded MMMs containing different zeolite topologies: NaA, NaX, and NaY, having a Si/Al ratio of 1.0, 1.23 and 54, respectively. Release studies showed that zeolites with a low Si/Al ratio interacted much more with drug molecules, allowing for a more linear release profile and a decrease in the release rate than the simple PDMS membranes. This behavior was more marked for membranes loaded with 5 wt % of the NaX zeolite and 2% of drug loading. In fact, after 6 h, they released just over 30% of the initial drug content, while membranes loaded with the NaA zeolite released about 67%. This phenomenon was attributed to the presence of supercages in the NaX zeolite into which the drug molecules entered and were consequently slowed down. Membranes with the hydrophobic NaY zeolite showed a burst effect [85]. In a different work, poly(ε-caprolactone) was used as the polymer matrix for producing electrospun MMMs containing the NaX zeolite and ibuprofen as the model drug [244]. They exhibited a better controlled membrane thickness, enhanced physical properties, a lower swelling ratio and higher release (about 80% after 8 h) in comparison with flat-sheet membranes prepared without zeolites (about 60%).

Regarding other inorganic fillers, an example of their use in MMM preparation comes from the production of electrospun silica/poly(ε-caprolactone)/polyethylene oxide fibers loaded with doxorubicin (dox), which were prepared for studying the effect of particle distribution in fiber mats on release properties [245]. The silica nanoparticles present in the dope solution for electrospinning tend to agglomerate on the external surface of the fibers. Ultrasonication of the samples before electrospinning resulted in a uniform distribution of the silical/dox in the fiber structure. After 33 days, this fiber mat presented a dox release of ca. 69%, higher than those obtained preparing fibers from sonicated polymer solutions. However, the creation of agglomerates can be favorably exploited to increase the release rate when the release of the drug is slow since a higher drug amount is exposed on the membrane surface. This approach was proved by introducing silica nanoparticles into electrospun fibers loaded with drugs, resulting in the creation of bumps when they are near the surface of the fibers [246].

Recently, nanostructured electrospun biodegradable PCL membranes were loaded with nano-hydroxyapatite and the gentamicin drug for treating periodontitis on Wistar rats [247]. Histological analysis demonstrated that the application of the nanostructured membrane provided a local antibiotic action and allowed for bone regeneration [247]. Transdermal patches loaded with curcumin and based on a chitosan/carboxymethylcellulose/akermanite composite matrix have been successfully produced for skin wound healing [248]. Tetracycline hydrochloride drug–nanoclay intercalates were incorporated into polyurethane electrospun nanofibrous membranes for wound healing [249]. The presence of the drug–clay intercalates in the polyurethane-fibers drastically decreased the contact angle, owing to the hydrophilic clay mineral character, thus leading to a rapid absorption of wound exudates, ensuring rapid healing of the skin. In vitro release showed a burst release profile in the nanofibers containing only the drug, while those containing drug–clay intercalates showed a sustained release profile for about 10 days with beneficial actions of wound healing. The drug release behavior was non-Fickian and promoted by concentration gradience and ion exchange [249].

The use of pectin as the base polymer for producing films containing drug carriers like inorganic nanoparticles is attractive for its safety [250,251]. Pectin grafted with the copolymer of 2-acrylamido-2-methyl-1-propanesulfonic acid and an acrylamide-based film loaded (via adsorption) with silver nanoparticles exhibited excellent adsorption and transdermal release behavior of the anti-Alzheimer’s drug donepezil [251]. The presence of nanoparticles allowed for an increase in film resistance to breakage by over 300 folds. The release mechanism followed zero-order kinetics and a non-Fickian type of diffusion. Additionally, the presence of silver nanoparticles (well known for their antimicrobial activity) conferred antimicrobial activity against Gram-positive and Gram-negative bacteria to the membranes. In a previous work, the same authors demonstrated the release attitude of graft copolymer pectin-based films, owing to the presence of zinc oxide nanoparticles as transdermal drug delivery vehicles [252]. The anticancer activity against the A431 skin cancer cell line of PVA nanofibers loaded with gold nanoparticles and curcumin was also demonstrated [253]. Gold nanoparticles have very low toxicity and small dimensions and can easily penetrate the skin without causing damage [232]. Other studies have been focused on the production of nanofibers based on PVA containing titanium oxide as the vehicle for releasing the vitamin B2 [254] and on the antibacterial action and wound-healing activity of SA-based membranes loaded with silver nanoparticles and hyaluronic acid [255].

It must be highlighted that the size of the fillers plays a crucial role in determining the drug delivery rate. When used as dug carriers, smaller particles can achieve higher loading due to the high surface area. As a consequence, increased therapeutic efficacy can be achieved [256]. However, very small particles may undergo a too rapid release (burst effect) and their tendency to agglomerate makes the fabrication of nanocomposite materials very challenging. In particular, taking into account that the horny SC layer has intercellular spaces in the range of 50–100 nm, smaller particles can have an enhanced percutaneous penetration capacity with potential toxic effects [257]. Therefore, their incorporation into the membrane matrix of nanoparticles with different sizes can modulate the release profile.

#### 7.2.2. Nanocomposite Membranes with Organic Fillers

The concept of mixed-matrix membranes can be extended by incorporating organic nanoparticles into a polymeric membrane matrix in drug delivery application [53,231,232]. One example is the production of transdermal patches based on a blend of ethyl cellulose and PVP in a 3:2 ratio with polyvinyl alcohol that were fabricated enclosing solid lipid nanoparticles (SLNs) that were prepared by the hot melt-homogenization method and loaded with the antidepressant paroxetine [258]. The drug encapsulation into the SLNs produced patches with higher drug permeation through the skin compared to simple transdermal patches and a sustained-release behavior. In a different work, a non-toxic and low-cost biomaterial double-layer biomembrane for dual drug delivery for the treatment of wounds has been developed by Oliveira et al. [259]. The membrane was based on a layer of chitosan/hydroxypropyl methylcellulose loaded with lidocaine and a second layer of sodium alginate nanoparticles loaded with the antibiotic polymyxin B sulphate. Fourier-Transform Infrared Spectroscopy, thermal analysis, and X-ray diffraction evidenced a strong interaction between the drugs and the functional groups of respective polymers. SEM analysis showed a uniform distribution of alginate nanoparticles on the membranes’ surface. The system exhibited good mechanical properties and was pH compatible for in vivo wound healing.

**Figure 11:**
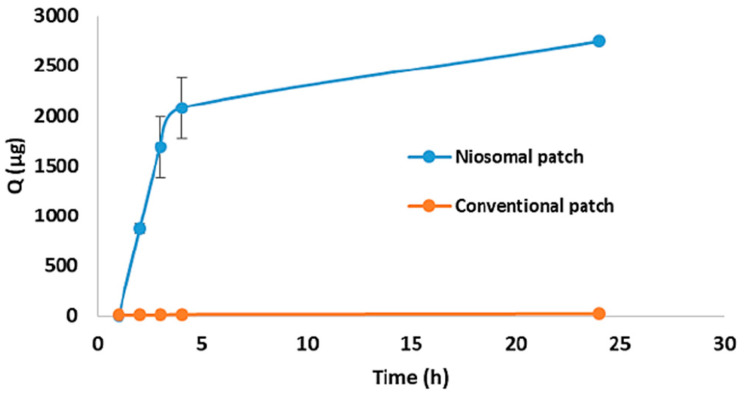
Cumulative release profile of clarithromycin for niosomal and conventional patches. Reprinted from ref. [262]. Open Access.

Eudragit RL 100 nanoparticles loaded with itraconazole have been fruitfully used as a carrier for enhancing transdermal permeation and the bioavailability of this antifungal drug. Their incorporation into PVA/HPMC-based membranes resulted in a biphasic release characterized by a fast drug release rate in the first 2 h and by a gradual release rate reduction over the subsequent 12 h [260]. By varying the ratio of PVA/Eudragit/HPMC, different stable and safe patches with enhanced skin permeation ability and those suitable for prolonged action were obtained [260]. In a different case, SLNs were employed as drug carriers in formulating rivastigmine tartrate Eudragit transdermal films for the treatment of Alzheimer’s disease [261]. A possible combination of the formulation components was strategically assessed with the aid of the full factorial Design of Experiments (DoE) method. In vitro and ex vivo release studies have demonstrated that by appropriately varying the parameters affecting the release rate (e.g., drug and SLN loading, as well as polymer and plasticizer concentration), a desired release profile could be obtained. Among the more recent applications of organic fillers, it is worth mentioning the preparation of a patch embedded with clarithromycin-loaded niosomal nanovesicles [262]. HPMC was used as the polymer matrix to form the membrane, while the plasticizer was PEG 400. The encapsulation efficiency of niosomes reached 86%. The niosomal patch showed antibacterial activity toward *Staphylococcus aureus* ATCC 6538. Furthermore, in vitro permeation studies through the commercial Strat-M^®^ membrane to mimic the skin showed that the optimized niosomal patch exhibited a significantly higher cumulative release (see Figure 11). Results of this work highlight the role of non-ionic surfactants in niosomal formulations as permeation enhancers for the penetration of a drug through the skin. Niosomes are easily included in transdermal films; they have an excellent capacity to encapsulate hydrophilic, lipophilic and amphiphilic drugs. They exhibit superior stability and low costs than liposomes [262,263,264].

**Figure 12:**
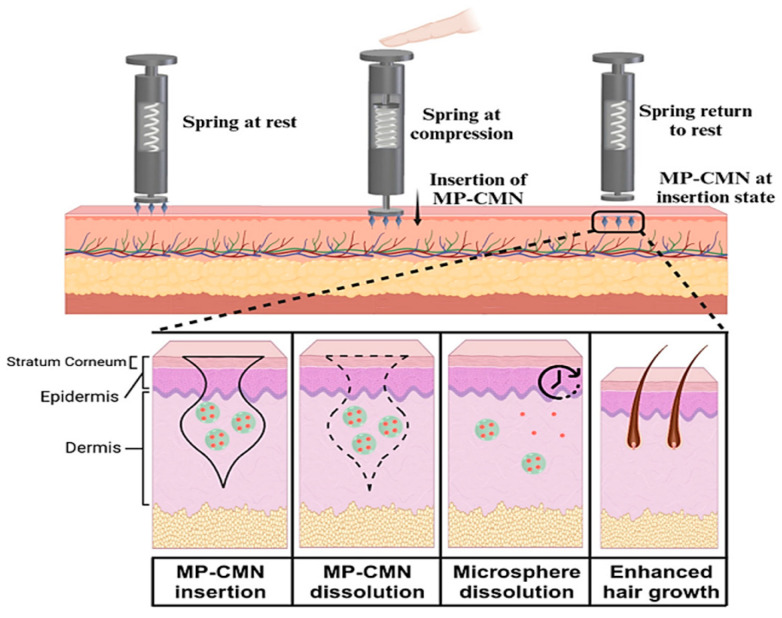
Representation of MP-CMN insertion via spring force application and sustained drug delivery into the skin. Adapted from ref. [266]. Open Access.

Water-soluble microneedle (MN) patches integrated with biodegradable minoxidil (MXD)-loaded microspheres of polylactic-co-glycolic acid (PLGA) have been also successfully prepared for long-acting hair regrowth treatment [265]. Upon the application of a patch on mouse skin microneedles immediately dissolved and delivered the drug-loaded microspheres into the skin. The latter acted as a drug reservoir for sustained release for 2 weeks to promote hair growth. Additionally, the mechanical penetration of microneedles through the mouse provided an additional stimulation to hair growth [265]. Recently, for the treatment of alopecia, a new non-adhesive patch based on candlelit microneedles was proposed. CMNs have incorporated PLGA encapsulating the NO-releasing PDE5 inhibitor TOP-M119 (M119), a vasodilator promoting hair growth [266]. CMNs are a kind of dissolving MN (DMN) that lack an adhesive layer and have a spring force applicator and a curve structure that locks them into the skin after insertion [266,267]. Figure 12 shows a schematic representation of alopecia treatment with the produced microneedle-based patch (MP-CMN) [266]. This strategy allows for the prevention of the microneedle from getting pushed out by skin resilience and tension, promoting extensive hair growth. Moreover, it allows for a reduction in application frequency in vivo and enables patient compliance [266,267].

### 7.3. Molecularly Imprinted Membranes in Transdermal Drug Delivery

Molecularly imprinted membranes (MIMs) are smart membranes having recognition sites complementary in chemical function, size and shape to a specific molecule of interest (called templates or print molecules) and are able of interacting with them in a specific way [268]. The sites can be located on the surface and/or within the membrane matrix, which exhibit selective binding and transport features. The production of MIM-based delivery systems allows for an increase in drug loading and its residence time into the macromolecular network, thus leading to a sustained release of the drug of interest. All these aspects lead to better control of the delivery process [6,64,269]. Additionally, in the presence of racemic drugs, the use of enantioselective-imprinted membranes allows for the delivery of only the pharmaceutically active enantiomer [6,270,271,272,273]. The successful exploitation of imprinted materials as advanced tools for controlled delivery objectives was first emphasized by Langer and Peppas [274], and the attention on this strategic approach has increased overtime, owing to the possibility of producing release systems with specific receptor sites for a given drug, thus miming the molecular recognition process occurring in biological systems [6,269,272]. Moreover, in comparison with biological receptors, these systems present the advantages of low cost and high stability, which render them attractive in many areas.

**Figure 13:**
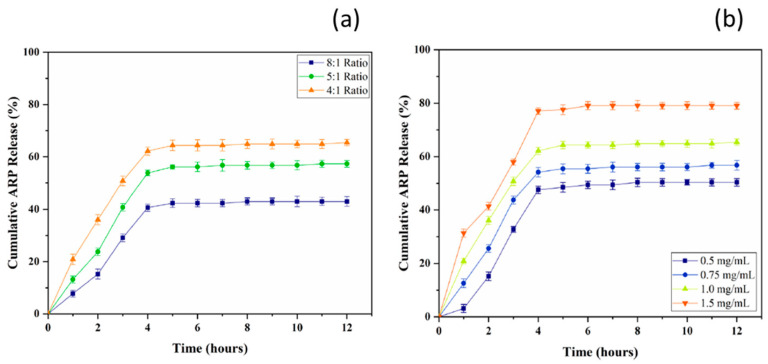
Cumulative release of ARP in 4:1, 5:1, and 8:1 molar ratios of HEMA/MBAAm (**a**) and different amounts of drug loads (**b**) of ARP-imprinted pHEMA cryogel patches. Readapted from ref. [282]. Open Access.

The application of imprinted membranes in controlled drug delivery approaches involves drug adsorption or its incorporation into the membrane matrix via the molecular interactions of drug-recognition sites. The selection of the more appropriate functional materials is influenced by the physiochemical properties of the therapeutic agent. This is an important aspect to also consider for hosting imprinted membranes with stimulus awareness in order to realize a remote control of drug release [275,276,277,278]. Regarding their application, one example is the transdermal delivery of the β-blocker S-propanolol from imprinted membranes prepared through different strategies [273,279,280]. Among them, in the last decade, Bodhibukkana et al. developed composite S-propranolol-imprinted membranes by the grafting-surface modification of a microporous cellulose support with a thin layer of S-propanolol-imprinted poly(methacylic) acid [279]. The obtained membranes exhibited good biodegradability and biocompatibility, as well as good enantioselective transdermal release through rat skin [279]. In a different approach, a chitosan gel formulation containing racemic propranolol was incorporated in a CA/imprinted poly(methacrylic acid) composite [273]. Released studies carried out on a rat skin confirmed that the incorporation of the original formulation into the imprinted membranes allowed for the controlled release of the two enantiomeric forms of propranolol. In fact, the developed imprinted membrane-based release system exhibited a facilitated release of the enantiomer template and achieved sustained drug release for over 48 h. After this time, 60% of the initial drug content was released from the system. The repulsive effect due to the negative charges present on chitosan and propranolol was also involved in the release rate behavior. On the other hand, the release of the opposite enantiomer was low. Additionally, the simple chitosan gel formulation releases the two enantiomers at the same extent. Finally, similar release systems prepared using poloxamer 407 as the reservoir-based polymer instead of chitosan exhibited a low non-enantioselective release rate (40% of the initial content), thus suggesting that the choice of the reservoir material used in patch formulation is an important parameter not to be underestimated [273]. Mitomycin C-poly(hydroxyethyl methacrylate-N-methacryloyl-(L)-histidine methyl ester)–Cu(II) [PHEMAH–Cu(II)] cyrogel membranes are promising for the sustained release of this anticancer drug [281]. In vitro release studies showed that the cumulative release of the drug decreased when increasing the amount of the crosslinker (methylene bisacrylamide, MBAAm). This behavior was attributed to an increase the rigidity of the crosslinked structure, owing to contraction and decrease in voids in the polymer network and to a reduction in macropore size [281]. In release studies carried out in PBS at pH 7.4 and 37 °C, the best operating conditions resulted in a polymer/crosslinker molar ratio of 1:4, an initial drug concentration of 200 µg/mL, and a polymerization temperature of −14 °C. Furthermore, imprinted membranes reached a loading efficiency of 70–80%, exhibited a sustained release of mitomycin C for over 150 h and resulted in non-cytotoxicity for the mouse fibroblast cell line L929 [281]. Very recently, 2-hydroxyethyl methacrylate (HEMA) and methylene bisacrylamide (MBAAm) at different molar ratios have been used for preparing cryogel MIMs using aripiprazole as the template drug to be administered via a transdermal route [282]. Aripiprazole is a drug used in the treatment of neurological disorders. The produced imprinted patches showed biocompatibility, and in their presence, cell viability of over 80% was observed after 48 h of treatment. Under basic conditions, the hydroxyl groups of HEMA undergo ionization and increased swelling, owing to an increase in the polymer’s capacity to adsorb water. The highest cumulative release was observed at pH of 5.5 and 7.4, reaching values of up to 80% of the initial drug loaded in the system (1.5 mg/mL) [282]. Figure 13a,b shows the cumulative release (%) vs. the HEMA/MBAAm molar ratio and as a function of pH, respectively [282]. All the experimental results confirm the suitability of these materials in biomedical applications and for transdermal delivery purposes. It was also established that the crosslinker ratio within the cryogel membrane structure affects the pore size and therefore the drug release rate [282].

Biodegradable mungbean starch/PVA films imprinted with the nonsteroidal anti-inflammatory drug sulindac were prepared via a UV curing process [283]. Starch-based biopolymers are attractive for transdermal delivery due to their low cost and easy processability. The release rate of sulindac from the prepared membranes was dependent on pH and temperature and was sustained for 20 days. In 2024, Zhang and co-workers [284] developed a chiral molecularly imprinted patch exploiting the synergistic effects of chiral molecularly imprinted polymers and pressure-sensitive adhesives for promoting the enantioselective controlled release of the non-steroidal anti-inflammatory drug (NSAIDs) flurbiprofen. The patch consisted of a chiral pressure-sensitive adhesive layer of L-N-acryloyl-phenylalanine (L-AP-PSA) and a module containing a molecularly imprinted polymer film (D-MIF) of p(L-N-acryloyl-phenylalanine (L-Aphe)) [284]. The patch was synthesized via click chemistry. The imprinted film adsorbed the R-isomer of the racemic drug loaded into the system, while the L-isomer was delivered from the adhesive layer [284].

Recent studies have also focused on the production of surface molecularly imprinted composite membranes for the enantioselective transdermal release of S-amlodipine [285,286]. For example, Men et al. [285] synthesized a thin S-amlodipine-imprinted layer of p(methacrylic acid) on the surface of PVA membranes via graft copolymerization. The crosslinking agent was N,N’-methylene bisacrylamide (MBA). The cumulative release was investigated in in vitro release studies performed with two-chamber diffusion cells, assessing the effect of permeation enhancers, DMSO, oleic acid and Azone (as a mixture or alone). Results evidence that the system with 1% of oleic acid alone in the presence of 0.9% of the thickener Carbopol 940 was the most efficient and allowed for the transport of S-amlodipine (2220.48 ± 138.72 μg/cm^2^) within 24 h, while with DMSO alone or with Azone, the transport was 2090.56 ± 199.49 μg/cm^2^ and 2150.56 ± 161.36 μg/cm^2^, respectively [285]. Carbopol 940 is a crosslinked polyacrylic acid polymer, having numerous carboxyl groups. At a concentration of 0.9%, it is in hydrated form and is melted into water to form a hydrogel. Therefore, when the hydrogel is completely hydrated, at this concentration, it has a percolated structure containing pools of solvent that promote the drug diffusion through the channels of the imprinted membrane [285]. MA and MBA were also used to develop an innovative S-amlodipine surface-imprinted membrane (S-ADP@SNP-SMIM) that was fabricated by also using methacrylic acid via synchronous grafting polymerization and crosslinked imprinting on the SiO_2_-NH_2_/PSF composite membrane substrate [286]. An important result was achieved with the production of melanin-containing cellulose-poly(methacrylic acid)-based MIP films for the controlled release of the antiarthritic drug methotrexate with the aim of reducing its side effects [287]. In binding studies, membranes selectively recognize methotrexate in the presence of the similar compound folic acid. The release rate of the membranes at acidic pH 5.5 was higher than the value observed at neutral pH. Additionally, upon laser irradiation at 808 nm, the release rate increased fast, owing to a physical change of the film. Another example of application is the production of polymeric natural rubber as the matrix-controlling system for the release of nicotine [288].

As it is easy to understand, imprinted membrane-based transdermal delivery systems involve the release of many drugs, preparation methods and polymeric materials. However, the real application of imprinted membrane-based release systems is still in its infancy, and further efforts are needed to achieve worldwide knowledge at the industrial level. In fact, their use is hindered by some challenges, like reproducibility biocompatibility and safety aspects of their preparation process, which entail the polymeric material, the solvent, crosslinker and waste disposal, operator risks, etc. [289,290]. For example, some issues to be solved are related to the excess of the porogenic solvent that is used in polymerization reactions [290]. According to the Green Chemistry Principles [291,292], preparation strategies involving the use of harmless solvents and reactants are strategic for developing safer release systems and rendering them suitable for technological transfer. From this viewpoint, a useful approach is the use of greener functional monomers and solvents like ILs and their more friendly derivatives, as well as DESs. Computational design also represents an important activity in the production of these systems, owing to the possibility of predicting intermolecular interactions and of identifying the more appropriate selection of chemicals, prioritizing safety and efficacy. Furthermore, a promising approach is the multi-drug imprinted strategy, which allows for a knock down in the consumption of reactants and waste generation. In addition, this strategy can have a role in the production of smart MIM-based devices (e.g., stimuli-responsive systems) for sequential sustained drug delivery in multitherapy. We are confident that the abetment of these barriers will help in their translation from the laboratory to the clinical scale.

## 8. Stimuli-Responsive TDDSs

**Figure 14:**
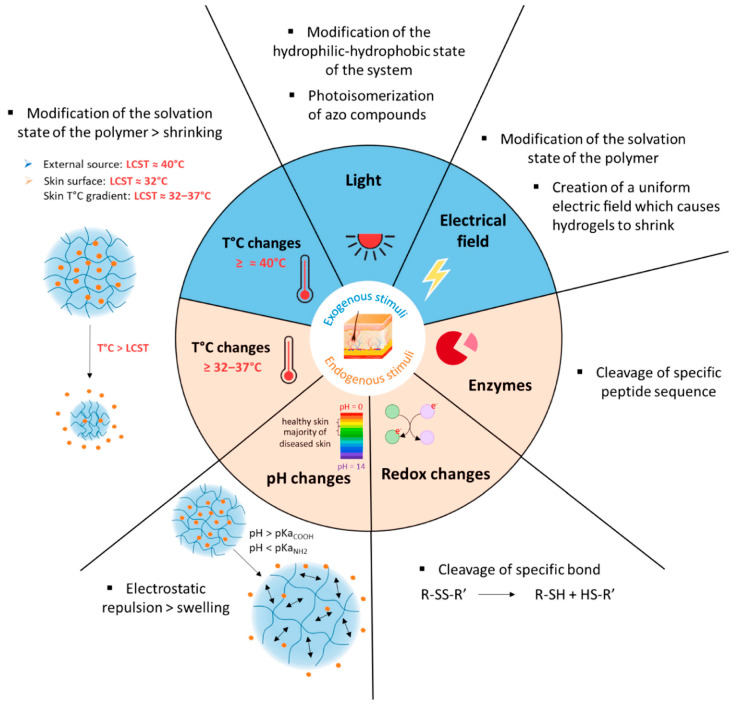
Overview of stimuli and their mode of action applied to the development of drug delivery systems for transdermal applications. Reprinted from ref. [297]. Open Access.

Triggerable drug systems represent a frontier for the TDDS field and are particularly interesting for smart biomedical applications [293,294]. In this respect, stimuli-responsive devices that react to external stimuli, such as heat, light, pH, ultrasound, or magnetic fields, are particularly interesting for smart biomedical applications, enhancing treatment specificity. The working principle of stimuli-responsive delivery systems is to deliver the drug in response to a variation of the microenvironment of sick tissue compared to normal tissue or in reaction to external stimuli. In this respect, stimuli can be distinguished as endogenous and exogenous stimuli. The first type includes internal variations of pH, enzymes, glucose levels, and reactive oxygen species. Exogenous stimuli include electric currents, heat, humidity, light, magnetic fields, mechanical forces, and ultrasound [293,294]. The responsive features can be provided by the polymer matrix or by the incorporated fillers [295,296]. Figure 14 illustrates the different stimuli and their mode of action applied to the development of drug delivery systems for transdermal applications [297]. Upon exposition at an electrical field, *electric current-responsive* delivery systems can undergo reversible redox reactions and structural changes that promote drug release. *Light-responsive* systems often contain photosensitive or thermosensitive agents with the ability to convert light into heat for regulating the release process. Magnetic field-responsive systems involve the presence of metal-based nanoparticles that can be guided by a magnetic field or convert the field’s energy into heat to control the release process. *Mechanical force-responsive systems* respond to a mechanical stimulus on the patient (in the muscles, tendons, bone joints, and other organs), generating structural changes under specific forces. *Ultrasound-responsive systems* utilize the acoustic energy generated by ultrasound stimulation to disrupt the polymeric structure, allowing for polymer dissolution and drug delivery. Instead, in humidity-responsive patches, the drug release is promoted by humidity variations, which allows for volumetric changes of the release system [293,294].

Finally, temperature-responsive membranes permit a precise control of the drug-release rate, responding to a temperature variation that can be due to physiological alterations (e.g., local inflammation or fever) or to an external heat stimulus to initiate or halt drug release. Among them, thermoresponsive membranes can be prepared using polymers that undergo a phase transition. For example, PNIPAM has a lower critical solution temperature (LCST) at around 32 °C. This material swells below this temperature and shrinks above it, thus modulating drug diffusion [293,298]. When the material shrinks, drug release increases. For example, poly(*N*-isopropylacrylamide) (PNIPAM) has been utilized as a thermosensitive polymer in drug delivery systems by grafting with other polymers or inorganic particles [299,300,301,302].

A pH-responsive surface-active IL (guanidinium oleate, [Gu][Ol]) was used to prepare mucoadhesive patches by mixing it with PVA [96]. The patch was loaded with 5-fluorouracil, an anticancer drug, achieving satisfactory transdermal delivery, reaching a 60% release of the loaded drug at pH 7.2 within 48 h.

Stimuli-responsive microneedle patches can respond to the physiological variations of our body or of the external environment, thus, achieving a continuous controlled drug release [303]. An interesting example is their application in the treatment of mouse brain glioblastoma (GBM), one of the most aggressive tumors [304]. In this context, a biodegradable silk fibroin microneedle (SMN) patch has been produced for bypassing the blood–brain barrier. It released spatiotemporally and sequentially multiple drugs to the tumor site upon activation by near-infrared (NIR) light irradiation and activated enzyme-induced silk protein degradation. The delivery system successfully regulated the tumor microenvironment and inhibited its volume growth for 4 weeks [304]. The combination of microneedles with poly(L-lactide-co-D,L-lactide) (PLA) as supporting array for producing implantable polymeric microneedles with a phototriggerable property was also exploited by Chen et al. [305]. The composite system released the analgesic lidocaine upon irradiation with NIR light that induced a phase transition in MNs. Variations of the irradiation allowed for a modulation of the delivery rate. More recently, NIR light-responsive nanofibrous membranes have been applied for treating skin bacterial wound infections [306]. Combating against bacterial infections is one of the great challenges for human health. Under NIR laser irradiation that produced hyperthermia, these membranes were capable of killing bacteria in the early stages of infected wounds, promoted collagen deposition and accelerated wound healing. They consisted of a PVA matrix containing tannic acid−Fe(III) nanoparticles as NIR laser-activated photothermal nanoagents and the herbal medicine berberine hydrochloride [306]. Another example comes from the production of the electrically pore-size-tunable polypirrole/dodecylbenzene sulfonate (PPy-DBS) nanoporous membrane electrocopolymerized on an anodized aluminum oxide membrane [307]. This material exhibits biocompatibility and a very large volume variation depending on the electrochemical state. So, the exposition of the electro-responsive membranes to an electric field determined a pore-size change of the membrane, allowing for a pulsatile release of the fluorescently labelled bovine serum albumin. In particular, at the oxidation state, the pores were opened and the release amount increased, while the closure of pores at the reduction state allowed for the stoppage of drug delivery [307].

Apart from the few cited examples, stimuli-responsive patches/transdermal release systems are also used in the treatment of diabetes, hair loss, heart diseases, obesity, etc. They are innovative tools enabling real-time remote regulation of the drug release profile [293].

## 9. Challenges and Outlooks

Over time, recent years have been marked by a relevant progress of CDDSs. This area is still the subject of extensive research studies and nano-based delivery technology is a prominent modern application. The most recent products are based on targeted therapeutic strategies and include injected nanocarriers, drug–polymer conjugates, new nanoparticle-based formulations, microneedle-based patches, etc. [308]. In order to achieve success and benefits of third-generation controlled release formulations, problems related to the previous release generations need to be solved. Some of them are the poor solubility and high molecular weight of some drugs, the use of non-toxic excipients, the control of the initial burst release, the drug delivery for a long time, the tumor targeting, and the passage through the blood–brain barrier [11,308,309]. Considering all these aspects, advanced drug delivery systems are required for overcoming these challenges and improve performance in clinical trials and the sustainability of CDDSs.

The researches and related strategies discussed in this review are contributing to the development of safe and highly efficient therapeutic tools to benefit society in the near future. However, some challenges related to clinical translation and commercialization need to be addressed. In particular, regarding TDDSs, despite their proven benefits, a continuous effort is required for their clinical translation and this concerns the conception of both more advanced materials and properly designed units. The main challenges are related to efficacy and safety for aligning them with regulatory legislation. The entire life cycle of the product should be considered, starting from the production of the materials applied in each formulation. In this respect, the use of sustainable materials and eco-friendly solutions is highly recommended [310]. Recent environmental directives and legislation have been appointed to regulate or in some cases ban toxic solvents in order to reduce their harmful emissions (e.g., the Reach Regulation [311]). A lot of work is ongoing to make more sustainable and eco-friendlier membrane-preparation processes in order to comply with the Green Chemistry Principles by implementing green solvents in the production of polymeric membranes [312,313,314]. Moreover, “soft” mechanochemical processing can be exploited to prepare nanocarriers for drug delivery (e.g., nanoparticles and nanocapsules) without using solvents [315].

The use of biopolymers may not necessarily be more environmentally friendly than petrochemical polymers, as demonstrated by Life Cycle Assessment (LCA) studies that estimate the impact of the production process from different perspectives. Indeed, the synthesis of a bio-based polymer can comprise unfavorable steps from an environmental point of view [316]. In this context, a comparative LCA study carried out on the production of polymeric membranes showed that the choice of solvent and polymer as well as the source of electricity are the major contributors to the environmental impact and process costs [314]. In general, the use of electricity obtained from renewable fonts can reduce the environmental impact. The use of a bio-based polymer (e.g., CA) can have either a positive (PVDF vs. CA) or negative (PSF vs. CA) environmental impact compared to other fossil-based polymers that depend on the production process of CA [314]. On the other hand, regarding the solvents used in polymer processing to obtain the final membrane, toxic solvents such as NMP have a negative environmental impact (40–60% of the total impact) [314].

Furthermore, plant-derived bioactive compounds with therapeutic benefits can be exploited in the prevention of oxidative and inflammatory diseases. However, challenges such as poor solubility, instability, and low bioavailability often hinder their therapeutic efficacy. DESs can also contribute to a reduction in the environmental impact of TDDSs.

Concerning nanocomposite materials, scale-up reproducibility and stability are important challenges limiting their application at the clinical level. Their synthesis still requires standardization to prevent the nanoparticles’ aggregation that typically happens due to strong intermolecular forces that can be achieved via a functionalization of the particles. No nanoparticle-encapsulated transdermal patch has attained FDA approval as yet [317]. Therefore, additional studies on the safety profile of nanocomposites are needed [318] to reach regulatory approval for use in humans of nanoparticle-based materials.

Stringent regulatory and manufacturing requirements are necessary for TDDS medical products. The introduction of in vitro human skin permeation experiments in the development of transdermal patches by the EMA guideline (MA/CHMP/QWP/608924/2014) on quality of transdermal patches [319] provided the possibility to rationalize in vivo experiments by reducing their number. However, in vitro/in vivo correlation models are necessary. For example, in vitro tests on human skin for the permeation of lipophilic drugs such as fentanyl has a weak correlation to in vivo outcomes when heat is applied [320]. An aspect to be considered is that the results of the in vivo preclinical studies carried out on mice are not always reproduced in clinical trials [308]. The integration of new technologies, including bioelectronics and AI, can provide valuable perspectives on the future of drug delivery design [294]. In this respect, theoretical modeling has an important role in predicting the interactions between biomaterials and biological systems [321]. Innovative technologies such as computational microscopy that uses machine learning to enhance traditional microscopy allow for a study of the interactions between nanoparticles and biological components with remarkable precision at the nanoscale level [322].

In the framework of microneedle-based patches, a collaborative endeavor including product developers, regulatory authorities, public health bodies (e.g., the World Health Organization and the United Nations Children’s Fund), and end-users was created in 2021 to accelerate the clinical translation of this technology applied to infancy vaccines [323]. The Regulatory Working Group is trying to identify and address key issues related to the production of microneedle array patches. Indeed, safety risks for such intradermal injection technologies have to be tackled.

Considering the end of life of medicated patches, a proper disposal of transdermal devices is essential in order to circumvent intentional abuse and/or misuse of the discarded patch adhesives [324,325]. Additionally, even though TDDSs are very simple to use and are patient-friendly, their administration requires proper controls to also avoid toxicity effects by bystanders, children and healthcare personnel [326]. Moreover, other health risks have to be considered, since some samples sold on the internet can contain trace elements, including those potentially toxic to humans [327]. To prevent land and water pollution, common guidelines and training in private hospitals, hospices and care homes are required for proper disposal [328]. The development of innovative reusable patches is greatly needed to reduce the accumulation of medical wastes [329,330].

It is important to highlight that TDDSs can be affected by the inter-individual variability of the patients that leads to different responses to the therapeutic treatment. Some studies are considering modeling to predict the coefficient of variation (CV) of the maximal plasma concentration after patch administration (Cmax). CV is inversely correlated with the drugs’ molecular weight and lipophilicity in the range of 200 < MW < 400 and 1.6 < log Koct < 4.3, respectively [331]. Moreover, modern technology allows for the creation of an individualized digital twin of a patient to adjust the therapy, as demonstrated for transdermal fentanyl release for chronic pain management [332]. A virtual set of patients was created by using Markov chain Monte Carlo (MCMC) that was based on actual patient data. In particular, the thickness of the skin layer was modified for each patient, considering gender, weight and age. In this way, the assisted therapy decreased the average pain intensity (by 16%) compared to conventional therapy, and the median time without pain passed from 23 h to over 72 h. Therefore, interdisciplinary studies with the aid of computational modeling can be very helpful in tackling this problem. In vivo studies on people from different genetic backgrounds, considering both pharmacogenetics and epigenetic factors, will provide greater insight into the underlying mechanisms of inter-individual variability in drug metabolism and response. This is also relevant for assessing personalized therapy by predicting efficacy and eventual side effects.

**Figure 15:**
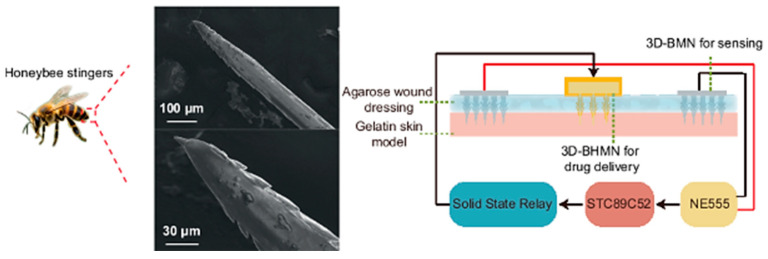
Three-dimensional-printed barbed microneedle patches combining biosensing and drug delivery in chronic wound management. Reprinted from ref. [335]. Open Access.

The use of innovative production methods such as additive manufacturing (e.g., 3D printing) can be further exploited for preparing innovative nanocomposites with a layered structure or miniaturized devices [333]. In fact, advanced percutaneous therapeutic devices such as engineered microneedle patches or microarray patches can be produced by high-precision 3D printing [334]. An interesting recent example is the fabrication of monolithic barbed microneedles (3D-BMN) for wound impedance sensing and barbed hollow microneedles (3D-BHMNs) for drug delivery mimicking honeybee stingers [335]. They have been fabricated with 3D printing (projection micro-stereolithography), combining painless transdermal biosensing and drug delivery (Figure 15). In particular, metal nanoparticles (e.g., Ag and Au) provide electrochemical performance, allowing for the monitoring of the wound status. The system enables the on-demand delivery of therapeutic agents modulated by the dynamic wound status that can be deduced by the sensed impedance value.

The combination of sensing and drug delivery enables the coupling of diagnostics and therapeutic treatment and is an interesting strategy with a broad translational potential. Synergistic therapeutic effects can be obtained by properly combining active ingredients. However, in this case, experimental studies are required to assess how the release rate of the drugs changes when they are used in combination compared to their separate loading [336,337].

## 10. Conclusions

In the framework of the different release systems, the attention of this review was mainly focused on the production of TDDSs. In fact, transdermal drug delivery is a successful strategy for supplying drugs and avoiding the typical problems related to oral dosage forms, such as gastric side effects and first-pass metabolism. Therefore, this topic was the focus of several studies in the last few decades with the aim of obtaining highly efficient release systems, moving from the macroscale to the nanoscale. These efforts have led to the development of advanced membrane-based formulations. Their multidimensional design is based on an interdisciplinary approach joining pharmaceutical and biological sciences, polymer chemistry, materials science, and engineering. All these disciplines can benefit from AI, which has a strong potential for accelerating the development of customized therapeutic solutions for a larger range of pathological diseases.

Polymeric materials have a key role in the formulation of TDDSs. In order reduce the dependence on fossil resources and environmental pollution, more sustainable materials are increasingly investigated. Thus, the more recent research studies deal with innovative biocompatible and stable polymeric materials with a special focus on bio-derived materials for preparing TDDSs. Moreover, natural compounds are routinely investigated as chemical enhancers or plasticizers or therapeutic agents.

The release kinetics can be predicted by different mathematical models. Some parameters affecting drug release are the membrane structure and configuration, membrane swelling, the hydrophilic/hydrophobic properties of the polymer-forming membrane and of the drug, the degradation/dissolution rate in the case of dissolving systems, the interaction between the polymer matrix and the drug, drug loading, the physiochemical properties of enhancers and plasticizers, the occurrence of external or internal stimuli, etc. Knowledge of the effects of these parameters on the release mechanism enables the development of the most suitable devices for a specific treatment. Molecular modeling has a key role in supporting research by providing insight into the underlying mechanisms of action of the components present in each formulation. Moreover, a valuable contribution can be obtained by computational modeling, allowing us to estimate individual drug effects, thus addressing inter-individual variability.

TDDSs based on organic, inorganic and hybrid nanoparticles as drug carriers are effective systems for better controlling the drug release behavior. Suited nanoparticles acting as reinforcement allow the use of biopolymers for the device fabrication. Also, the development of imprinted membranes exhibiting specific recognition and transport properties has a great to produce smart release systems highly specific towards targeted drugs.

Well-known preparation processes are available for the fabrication of the drug-loaded membranes permitting a fine morphology control that is strictly linked to the release properties. Furthermore, innovative technologies including electrospinning and 3D-printing are available to produce such devices. In addition, the use of minimally invasive nanoarchitectures such as microneedles and engineered electrospun membranes offers interesting perspectives for achieving improved performance.

The incorporation of multi-drugs for combined therapies and the use of nanomaterials with biocompatible and biodegradable multifunctional components enable personalized therapeutic treatments. The demonstrated combination of sensing and drug delivery enables us to couple diagnostics and therapeutic treatment and is a fascinating strategy with broad translational potential. Additionally, stimuli-responsive patches are highly suitable for on-demand drug delivery.

However, some challenges related to clinical translation and commercialization of future drug delivery tools need to be addressed. They include the limited availability and process complexity of naturally derived polymers, the conception of common guidelines for both the production and safe disposal of such devices, in vitro/in vivo correlation models, deeper knowledge of the mutual interactions of the components in the formulation, studies on the safety profile of nanoparticles used in the formulations, modulation of the therapeutic treatment based on the sickness grade, and a reduction in the incidence of inter-individual variability.

The integration of new technologies, including bioelectronics, can assist in the design of safer innovative delivery devices.

## Figures and Tables

**Table 1:** Kinetic models commonly used to describe drug release.

Model	Mathematical Equation	Note
Zero order	Q = Q_0_ + k · t	- Drug dissolution in some transdermal systems, matrix tablets with low soluble drugs, coated forms, osmotic systems, etc. [34]. - Q_0_ is the initial concentration of drug released (usually, Q_0_ = 0).
First order	Q = Q_0_ exp (−k · t)	- Describes the release of water-soluble drugs [35].
Higuchi	Q = k_H_ · t^1/2^	- Transdermal systems and matrix tablets containing water-soluble drugs [36]. - Assumptions: the initial drug concentration in the system is above its solubility limit, diffusion takes place only in one direction, diffusion is constant, the drug size is smaller than the release matrix thickness, and swelling and dissolution of the polymer matrix is insignificant.
Hixson–Crowell	Q^1/3^ − Q_0_^1/3^ = k · t	- Pharmaceutical dosage forms, such as tablets, where dissolution occurs in planes parallel to the drug surface, result in constant geometry during time [32].
Korsmeyer–Peppas	Q/Q_0_ = k · t^n^	- Semi-empirical model [31]. - Release from polymeric systems having different geometries (sheets, cylinders, spheres, disks and polydisperse microspheres). - *n* < 0.5: Fickian release similar to Highuchi’s model; - *n* = 0.5: pure diffusion Fickian controlled drug release; - 0.5 < *n* < 1: a combination of the erosion of the polymeric chains and of pure diffusion occurs (anomalous); - *n* = 1: the release is governed by swelling-controlled drug release or by erosion (non-Fickian) [37,38].
Weibull	Q/Q_0_ = 1 − exp [(−b ∙ t^a)]	- Empirical model [32].
Baker–Lonsdale	[1 − (1 − Q/Q_0_)^2/3^] ∙ Q/Q_0_ = 2/3 ∙ k · t	- Release data from several formulations of microcapsules or microspheres [39,40].
Hopfenberg	Q/Q_∞_ = 1 − (1 − k · t)^n^	- Drug release from erodible polymers and various geometrical forms [31]. - k = k_0_/(C_0_ a_0_) - k_0_ is the erosion grade constant, C_0_ is the initial concentration of the drug in the matrix, and a_0_ is the initial radius of the sphere or cylinder or the half film thickness; - *n*: 1, 2, or 3 for film, cylinder, or sphere.
Gompertz	Q/Q_0_ = exp [α · exp(β · logt)]	- Used to define in vitro dissolution profile [32]. - Drugs having good solubility and intermediate release rates. - α is a scale parameter that determines the undissolved proportion at time t = 1, and β is a shape parameter representing the dissolution rate per unit of time.
Bhaskar	−Log(1 − Qt/Q_∞_) = B · t^0.65	- Describes the diffusion of a drug from resins and inorganic particles [41]. - B is the kinetic constant.

Q represents the amount of drug released at time t, Q_0_ is the initial amount of the drug, Q_∞_ is the amount of the drug released after an infinite time, and k is the release constant.

**Table 2:** Polymeric materials used in TDDSs, classified on the basis of their origin.

Polymer Name	Strong Points	Weak Points	Ref.
Synthetic			
Crosslinked polyacrylic acid (carbopol)	Excellent bio/mucoadhesion and pH responsive.	Acidic nature of the gel can result in irritation and damage (e.g., to eye tissues).	[74,75]
Ethylene vinyl acetate (EVA)	Biocompatibility, transparency, and heat processible. Many properties of EVA can be very easily varied via the VA content.	Limited drug loading capacity of hydrophilic drugs due to its high hydrophobic nature; not biodegradable.	[76,77]
Polyaspartamide	Excellent biocompatibility and biodegradability. Derivatives such as Poly(N-isopropylacrylamide) (PNIPAAm) are temperature-responsive polymers with a lower critical solution temperature (LCST) at around 32 °C.	Insufficient mechanical strength.	[78,79]
Polycaprolactone (PCL)	Biodegradability and biocompatibility; miscible and mechanically compatible with many other polymers.	Low mechanical strength and slow biodegradation rate.	[80,81,82,83]
Polydimethyl siloxane (PDMS)	Excellent optical, electrical and mechanical properties; biocompatibility; transparency; resistance to biodegradation.	Hydrophobic (resulting in the adsorption of proteins from the surrounding biological environment); hydrophilized PDMS surfaces tend to recover their native hydrophobic state within a few minutes.	[84,85,86]
Polyethylene oxide (PEO)	Biocompatibility, thermoplastic, hydrophilic and water solubility.	Hypersensitivity.	[87]
Polyether block amide (Pebax)	Flexibility (enabling plasticizer-free formulations), strength, biocompatibility, and tunable properties by changing the ratio of the copolymer blocks.	Poor water absorption.	[88,89]
Polyglycolic acid (PGA)	First synthetic biodegradable polymer.	Difficult to process; rapid degradation.	[90]
Poly(lactic acid) (PLA)	Biodegradability (ability to decompose into non-toxic components under industrial composting), biocompatibility; more cost-effective and widely available compared to other biodegradable materials, such as PVP or PVA; used in the fabrication of dissolving microneedles. It can be derived from 100% renewable bio-resources (e.g., rice, wheat and sweet potato) through fermentation and polymerization.	Low degradation rate; acid degradation by-products; poor impact strength; occurrence of “burst release” in PLA-based systems.	[91]
Poly(lactic-co-glycolic acid) (PLGA)	Biodegradability and biocompatibility. It degrades through hydrolysis in the body, generating lactic and glycolic acids that are natural by-products involved in metabolic processes. Solubility in a wide range of common solvents (e.g, chlorinated solvents, tetrahydrofuran, and acetone). Degree of crystallinity, melting point, and mechanical strength can be changed by choosing the right molecular weight of the polymer.	Production and scaling difficulties; occurrence of “burst release” in PLGA-based systems.	[92,93]
Polymethacrylates (Eudragit)	Elasticity, self-adhesive, good adhesion to the skin and transparency.	Significant stickiness encountered during manufacturing.	[94,95]
Polyvinyl alcohol (PVA)	Biocompatibility and toxicologically safe.	Limited mechanical strength and high level of porosity in hydrogel.	[96,97,98,99]
Polyvinyl butyral (PVB)	Transparency.	Brittleness.	[100]
Polyvinyl pyrrolidone (PVP)	Non-toxic, temperature resistant, pH stable, biocompatible, and biodegradable.	Allergic reactions; highly hygroscopic (tough to store and handle) tackiness of the prepared films.	[98,101,102]
Semi-Synthetic			
Cellulose (ether) derivatives: - Carboxymethyl cellulose (CMC); - Ethyl cellulose (EC); - Hydroxy propyl cellulose (HPC); - Hydroxy propyl methyl cellulose (HPMC).	Non-toxicity, biocompatibility and water solubility. Ability to take up water from mucus, resulting in adhesive properties for buccal, ocular, vaginal, nasal and transdermal formulations. HPC or EC greatly expand the solubility of poorly soluble drugs by forming amorphous solid dispersions.	Low solubility, as well as low mechanical strength and thermal stability; high hydrophilicity.	[103,104]
Cellulose (ester) derivatives: Cellulose acetate (CA)	Good solubility in common solvents, which is different from insoluble cellulose; derived from abundant cellulose sources.	Rigid and brittle, requiring plasticizers.	[80,105]
Chitosan	Biocompatibility, biodegradability, low immunogenicity. and low toxicity; high swelling capacity and low production costs.	Potential batch-to-batch variability; poor solubility; inferior mechanical properties and brittleness (especially for microneedles).	[106,107,108]
Natural			
Agar (mixture of agarose and agaropectin)	Low cost and readily forms gels.	Requires heat to dissolve in water.	[83,109]
Collagen	Low immunogenicity and easy production of films, sponges and particles.	Potential risks of pathogen transmission for animal-derived collagen.	[110,111]
Hyaluronic acid (HA)	Excellent biocompatibility (a major component of the extracellular matrix), biodegradability and non-immunogenicity.	Rapid biodegradability.	[112,113,114]
Guar gum	Safe, biodegradable and water soluble; good film-forming ability.	Soluble only in water; high swelling capacity, which requires derivatization, grafting and network formation.	[115]
Natural rubber	Non-toxic, biodegradable, and cost effective and obtained from renewable natural resources; elasticity; flexibility.	Hydrophobicity, which requires blending with polar polymers	[116,117]
Pectin	Natural availability, biocompatibility, biodegradability, non-toxicity and low cost. Its use valorizes agro-food waste.	Brittleness; strong hydrophilicity, leading to excessive water absorption and potential instability, affecting consistent drug release.	[118,119]
Sodium alginate (SA)	Biocompatibility, biodegradability, non-toxicity; low cost of extraction and processing; good hydrogel-forming ability. Chemical modifications can be exploited to fabricate materials that respond to external stimuli.	It is difficult to produce electrospun mats due to the high number of hydrogen bonds and the high viscosity.	[109,120,121]
Xanthan gum	The presence of hydroxy and carboxy groups allows for chemical modification with the aim of improving physicochemical properties (e.g., mechanical and thermal stability, solubility, and swelling).	Microbial contamination, high viscosity, poor shear resistance, inadequate mechanical and thermal properties and uncontrolled rate of hydration.	[122]

**Table 3:** Chemical enhancers used in TDDSs.

Molecule Type		Mechanism	Ref.
Alcohols		Lipid extraction in the *stratum corneum*, resulting in increased water in the lipophilic region between layers. Ethanol and isopropanol can accumulate within the hydrophilic domain, thus increasing the solubility of drugs in this region.	[123,124]
Surfactants		Modifies the protein structure of the SC. Disrupt the lipid matrix. Increase the solubility and diffusivity of active compounds.	[125]
Cyclic structures	Sulphoxides	DMSO disrupts lipid organization and may displace protein-bound water.	[126,127]
	Cyclic ureas	Increase water content in the SC. Keratolytic activity.	[128]
	Amides (e.g., Azone^®^)	Highly lipophilic and can disrupt lipid packing.	[62]
	Pyrrolidone derivatives	Interact with the keratinized region of the SC and alter the solubility properties of the SC.	[129]
Terpenes		Disrupt the lipid matrix layer of the SC.	[130]
Essential oils		Disintegration of the highly ordered intercellular lipid structure between corneocytes in the SC. Interaction with intercellular domains of proteins, inducing their conformational modification. Increase drug partitioning.	[131]
Herbal extracts		Lipid disruption. Protein interaction. Enhanced drug solubility.	[132,133]
Ionic liquids		Modification of the lipid barrier in the SC, thus facilitating drug diffusion.	[134,135,136]

**Table 4:** Some commercial transdermal patches and their unique features (adapted from ref. [58]).

Drug	Indication	Product Name	Duration of Application	Ref.
Asenapine	Mania and bipolar disorder	Secuado^®^	24 h	[177,178]
Bisoprolol	Atrial fibrillation	Bisono^®^	24 h	[179]
Buprenorphine	Management of pain	Butrans^®^ Transtec^®^	7 days	[180,181]
Capsaicin	Peripheral neuropathic pain	Qutenza^®^		[182]
Clonidine	Hypertension, tic disorder, Tourette’s syndrome, attention deficit, and hyperactivity disorder (ADHD)	Catapres-TTS^®^	7 days	[183,184]
Dextroamphetamine	ADHD	Xelstrym^®^	Up to 9 h	[185]
Diclofenac diethylamine	Inflammation	NuPatch^®^		[186]
Donepezil	Alzheimer’s disease	Adlarity^®^	7 days	[187]
Estrogen	Postmenstrual syndrome	Fematrix^®^	7 days	[188]
Ethinyl estradiol	Prevent pregnancy	Ortho Evra^®^	7 days	[189]
Fentanyl	Moderate/severe pain	Duragesic^®^ Matrifen^®^	72 h	[190,191]
Granisetron	Anti-emetic	Sancuso^®^	Up to 7 days	[192]
Levonorgestrel and estradiol	Postmenstrual syndrome	Climara Pro^®^	7 days	[193]
Lidocaine	Treatment of pain	Lidoderm^®^ Dermalid^®^	Up to 3 times daily for no more than 12 h	[194,195]
Methylphenidate	ADHD	Daytrana^®^	Up to 9 days	[196]
Menthol and methyl salicylate	Muscle and joint pain	Salonpas		[197]
Nicotine	Smoking cessation	Habitrol^®^ Nicoderm^®^ Nicoderm CQ^®^ Nicorette^®^	24 h 16 h	[198,199]
Nitroglycerin	Angina pectoris Relieves pain after surgery	Deponit^®^ Nitro-dur^®^	12–14 h	[200,201]
Norethindrone estradiol	Symptoms of menopause	Combipatch^®^	3–4 days	[202]
Oxybutynin	Overactive bladder	Oxytrol^®^	3–4 days	[203]
Rivastigmine	Alzheimer’s disease	Exelon^®^	24 h	[204]
Rotigotine	Parkinson’s disease	Neupro^®^	24 h	[205]
Selegiline	Depression	Emsam^®^	24 h	[206]
Scopolamine	Motion sickness	Transderm-scop^®^	72 h	[207]
Testosterone	Hypogonadism	Androderm^®^	24 h	[208]

## Data Availability

No new data were created or analyzed in this study.
